# Parabrachial *Calca* Neurons Influence Aversive and Appetitive Taste Function

**DOI:** 10.1523/ENEURO.0191-25.2025

**Published:** 2025-10-07

**Authors:** Christian H. Lemon, Jinrong Li, Md Sams Sazzad Ali, Neville M. Ngum, Kyle T. Zumpano, Catori J. Roberts

**Affiliations:** School of Biological Sciences, University of Oklahoma, Norman, Oklahoma 73019

**Keywords:** behavior, *Calca*, CGRP, mouse, parabrachial, taste

## Abstract

The parabrachial (PB) nucleus participates in taste processing and integration with other senses. PB neurons that express the *Calca* gene support sensory-integrative responses, albeit only limited data have addressed their influence on taste. Here we investigated how chemogenetic dampening of PB-*Calca* neurons affected mouse orosensory preferences for diverse taste stimuli in brief-access fluid exposure tests, which capture oral sensory/tongue control of licking behavior. Intracranial delivery of Cre-dependent viruses in female and male *Calca*^Cre/+^ mice induced expression of the inhibitory designer receptor hM4Di:mCherry (hM4Di mice) or fluorophore mCherry alone (mCherry control mice) in PB-*Calca* neurons. Several weeks later, hM4Di and mCherry mice entered brief-access tests where they could lick solutions during discrete, seconds-long trials. Stimuli included concentration series of the behaviorally avoided bitter taste stimuli quinine and cycloheximide, the appetitive sugar sucrose, and mildly cool water and less preferred innocuous warm water. Blinded experimenters administered the hM4Di ligand clozapine-*N*-oxide (CNO) to all hM4Di and mCherry mice prior to daily tests. With CNO, hM4Di mice displayed greater average licking (i.e., less avoidance) of quinine than mCherry mice (*p* < 0.05). Moreover, male hM4Di mice selectively showed reduced mean licking preferences for sucrose under CNO (*p* < 0.05). These data suggest that PB-*Calca* neurons participate in both aversive and appetitive taste-guided behaviors, with their role in appetitive taste dependent on sex. Finally, orosensory responses to cycloheximide and thermal-controlled water did not differ (*p* > 0.05) between hM4Di and mCherry mice under CNO. Results are discussed considering functional differences among stimuli and study limitations.

## Significance Statement

Parabrachial neurons that express the *Calca* gene are implicated in protective responses evoked by multiple senses, including taste. Using Cre-directed chemogenetics and lickometry assays in mice, we found evidence that parabrachial *Calca* neurons may support broader, more diverse functions in gustatory processing. Chemogenetic suppression targeted to these cells reduced licking avoidance of the bitter taste stimulus quinine and also decreased licking preference for the preferred sugar (sweet) sucrose, with the latter effect arising exclusively in male mice. Our results agree with recent studies reporting involvement of parabrachial *Calca* neurons in both aversive and appetitive sensory valence coding and suggest there is a sex dependence to their role in appetitive taste.

## Introduction

Taste participates in the recognition of nutrient and toxin chemicals in potential food sources. In mammals, these chemicals engage diverse families of taste receptor proteins expressed by receptor cells comprising taste buds on lingual and intraoral surfaces (Roper and Chaudhari, 2017; Kinnamon and Finger, 2019). Gustatory neural information is relayed to the brain by cranial nerve fibers that terminate in the rostral nucleus of the solitary tract (rNTS) in the medulla (Pfaffmann et al., 1961; Halpern and Nelson, 1965). In rodents, the rNTS houses neurons that project ascending gustatory signals to the parabrachial (PB) nucleus of the pons (Norgren, 1974). The PB area is implicated with multiple autonomic and sensory roles and maintains neurons with axons that reach diverse brain regions associated with affect, including the amygdala and bed nucleus of the stria terminalis (Norgren, 1976; Saper and Loewy, 1980; Fulwiler and Saper, 1984; Gauriau and Bernard, 2002; Chiang et al., 2019).

In the PB area, neurons marked by the transcription factor Satb2 were implicated in appetitive and aversive taste function (Fu et al., 2019; Jarvie et al., 2021). Moreover, there is some evidence to suggest that PB neurons expressing calcitonin gene-related peptide (CGRP) encoded by the *Calca* gene participate in responses to aversive tastes (Jarvie et al., 2021; Kang et al., 2022; Kim et al., 2024b). PB-*Calca* neurons are implicated for roles in homeostasis and protective processing, including regulation of appetite and mediating pain-related responses (Carter et al., 2013; Campos et al., 2018; Palmiter, 2018). Notably, some PB-*Calca* neurons can respond to valence-aligned sensory inputs originating from more than one modality and across diverse bodily systems and receptive fields (Campos et al., 2018; Kang et al., 2022). This integrative feature may be expected of neurons that register the physiological value of a sensation rather than its location or identity.

*Calca* neurons densely populate lateral regions of the PB area identified, using neurophysiology and optogenetics, to contain taste-active cells that receive convergent input from ascending trigeminal somatosensory circuits (Li and Lemon, 2019; Li et al., 2022). Some of these taste-integrative neurons responded to both gustatory and intraoral trigeminal stimuli, activating to chemesthetic and thermal nociceptive inputs (trigeminal) and the bitter tastants quinine and cycloheximide (Li and Lemon, 2019; Li et al., 2022). Quinine and cycloheximide are ecologically and functionally diverse bitter-tasting chemicals/toxins that elicit concentration-dependent reductions in fluid licking (i.e., avoidance) in rodents (Boughter et al., 2005; Hettinger et al., 2007; Travers and Geran, 2009). Based on their location, PB taste-integrative neurons may include cells marked by the *Calca* gene. Relatedly, consumption of quinine activates a subset of PB-*Calca* neurons (Kang et al., 2022), with functional suppression of PB-*Calca* cells reducing ingestive aversion toward quinine in mice (Jarvie et al., 2021; Kang et al., 2022). However, the role of *Calca* neurons in orosensory responses to diverse bitter taste stimuli is unknown, with possible sex effects unaddressed.

Here, Cre-directed chemogenetics was used to study how temporary dampening of activity in PB-*Calca* neurons affected licking avoidance behaviors that mice show toward the bitter taste stimuli quinine and cycloheximide. Studies also examined how chemogenetic suppression of PB-*Calca* cells influenced mouse licking preferences for the preferred sugar sucrose. This work was accomplished by blinded experimenters testing a large number of experimental and control mice of both sexes in brief-access lickometry tests. Brief-access tests monitor rodent licking responses to fluids in short, seconds-long trials, which captures oral sensory guidance of ingestive preference while mitigating post-ingestive influences (Smith, 2001; Boughter et al., 2002).

Analyses revealed that during chemogenetic suppression targeted to PB-*Calca* neurons, female and male mice increased the number of licks they emitted to quinine (i.e., showed reduced quinine avoidance), with the magnitude of increase variable across animals. In contrast, licking responses to cycloheximide were unaffected. While there are discussed limitations to comparing these results, they associate with known functional differences between quinine and cycloheximide, including differential responding to these bitters by taste-active PB cells (Geran and Travers, 2009; Travers and Geran, 2009; Li and Lemon, 2019; Li et al., 2022). Furthermore, chemogenetic suppression directed to PB-*Calca* neurons reduced mouse licking preferences for sucrose, with these reductions showing significant sex dependence and emerging only in males. That perturbation of PB-*Calca* neurons affected both avoided (quinine) and preferred (sucrose) tastes associates with recent data on an ability of this cell class to bidirectionally encode appetitive and aversive stimuli (Kim et al., 2024a). Lastly, our results concerning participation of PB-*Calca* neurons with sucrose preference also provide evidence for a sex-specific effect of this brain cell-type on gustatory behavior.

## Materials and Methods

### Mice

All experiments and procedures were approved by the University of Oklahoma Institutional Animal Care and Use Committee and followed the *Guide for the Care and Use of Laboratory Animals* by the National Research Council. All studies herein used adult female and male *Calca*^Cre/+^ mice. These mice were generated by crossing homozygous *Calca*^Cre^ mice [strain #033168, The Jackson Laboratory (JAX); Carter et al., 2013] with C57BL/6J (B6) mice (strain #000664, JAX). A total of 88 *Calca*^Cre/+^ mice were surgically prepared and tested in these studies. At the onset of behavioral testing, study mice were 34.0 (mean) ± 12.8 (SD) weeks of age. On average, females (*n* = 47) weighed 23.4 ± 3.0 g whereas males (*n* = 41) weighed 31.5 ± 3.9 g.

Before surgery, mice were group housed in disposable cages with *ad libitum* access to standard mouse chow and filtered water. After surgery, each study mouse was singly housed in a disposable cage, with *ad libitum* food access but water availability regulated during behavioral studies, as below. Mouse cages were housed in an individually ventilated (HEPA filtered) racking system (Innovive) located in a climate-controlled room maintained on a 12 h light/dark cycle. Individual cages were prepared with standard bedding and enrichment materials (e.g., paper huts, cardboard tubes). Leading up to training for behavioral tests, bedding was changed by husbandry staff on a regular schedule. During experiments, bedding was changed by experimenters as needed to avoid housing disruptions.

### Surgery and bilateral intracranial microinjections

An intracranial microinjection procedure was used for viral delivery of fluorophore and DREADD (designer receptors exclusively activated by designer drugs) proteins to PB neurons. Mice were divided into experimental and control groups depending on the type of virus injected. Experimental *Calca*^Cre/+^ mice (hereafter referred to as hM4Di mice) received bilateral microinjections of a Cre-dependent virus that supported expression of the inhibitory Gi-DREADD hM4Di and fluorophore mCherry (AAV1-hSyn-DIO-hM4D(Gi)-mCherry, #44362, Addgene) selectively in *Calca*-positive neurons. When stimulated with CNO, hM4Di reduces neural firing (Urban and Roth, 2015; Roth, 2016), including in PB-*Calca* cells (Carter et al., 2013). Control *Calca*^Cre/+^ mice (hereafter known as mCherry mice) received bilateral microinjections of a virus supporting Cre-activated expression of mCherry alone (AAV1-hSyn-DIO-mCherry, #50459, Addgene) in *Calca* cells.

Over the course of these studies, groups of approximately nine mice, on average, were surgically prepared (one or two per day) and then, following recovery and a waiting period, tested together in behavioral studies as a squad. Ten squads were examined. Each squad included female and male mCherry mice and female and male hM4Di mice to avoid temporal confounds with testing different mouse groups/sexes.

All surgical tools were sterilized prior to use. The stereotaxic device (Model 1900 Stereotaxic Alignment System, Kopf Instruments) and surrounding workstation/countertop area were cleaned to prepare a sterile field. Approximately 30 min prior to surgery, mice received an injection of the antibiotic gentamicin (5 mg/kg, s.c.) to mitigate infection. Mice were then placed in a clear plexiglass rodent induction chamber and anesthetized with ∼3% isoflurane in oxygen, delivered at ∼1 L/min. Once anesthetized, the scalp was shaved free of fur, and mice were transferred to the stereotaxic head frame with their snout positioned in a gas anesthesia nose cone with incisor bar. Anesthesia was maintained by administering 1–3% isoflurane in oxygen, delivered at ∼0.6–1 L/min. The mouse was positioned atop a feedback-controlled heating pad maintained at 37°C. Lubricating eye (ophthalmic) ointment was applied to both eyes.

Anesthesia depth was monitored by absence of eyeblink, the absence of foot withdrawal following heavy pinch, a lack of startle from tail pinch, or monitoring for any other overt signs of response to physical stimuli. Once surgical-level anesthesia was ensured, an antiseptic (70% ethanol) followed by antibiotic (betadine) was topically applied to the bare scalp. A midline incision was made to expose the cranium. The skull was then brought into final alignment and leveled using stereotaxic measurements made from cranial fissures. Coordinates for targeting the microinjection needle tip to the PB area were as follows: 4.9–5.3 mm caudal of bregma, 1.0–1.3 mm lateral, and ∼2.6 mm ventral from the brain surface. These coordinates were obtained from published (Franklin and Paxinos, 2008) and online (Allen Brain Atlas) sources.

A sterilized drill bit was used to make a small craniotomy at the targeted location on the skull. A glass Hamilton microsyringe coupled to a sterilized 33-gauge beveled needle was then positioned perpendicular to the skull in a syringe pump (Micro 4 MicroSyringe pump, World Precision Instruments) coupled to the stereotaxic device. Based on coordinates, the needle tip was then slowly and precisely lowered into brain tissue to reach the PB area by using the fine-control stereotaxic manipulator arms, with tip position tracked using a digital readout.

After waiting for ∼10 min to allow brain tissue to recover from needle insertion, 0.5 µl of the virus was ejected from the syringe needle tip at a pump-controlled rate of 0.05 µl/min. Once the injection was complete, the needle remained in place for an additional 5–10 min before being slowly withdrawn dorsally from the brain and skull using fine controls on the stereotaxic device. The craniotomy and microinjection procedures were repeated for the contralateral PB nucleus.

Prior to closing the incision site, bone wax was applied to the craniotomies to seal them. One drop of the long-lasting local anesthetic bupivacaine was applied to the skin and periosteum surrounding the craniotomy. The scalp incision was closed by silk suture. Mice were then removed from the stereotaxic device, hydrated with 0.5 ml lactated ringers (s.c.), and administered buprenorphine (0.05–0.2 mg/kg, i.m.) for management of potential discomfort or pain.

Once mobile after surgery, each mouse was singly housed and monitored daily until it fully recovered from the procedure. Mice typically showed normal ambulatory behavior the day after surgery. Sutures disappeared after several days. No mice required additional analgesics during recovery. Mice entered behavioral tests >8 weeks following surgery.

### Experimenter blinding

Prior to behavioral testing, a lab partner matched a unique alphanumeric code to each study mouse and randomized their cage locations in the colony housing rack before behavioral training commenced. The codes were used to label mice in lieu of all other identifying mouse information, which blinded the experimenters handling and running mice, including administering CNO, to mouse DREADD group (i.e., hM4Di or mCherry). All mice had black coats and indistinguishable outward appearances, facilitating blinding. Blinding was used to collect behavioral data across all mouse training and test sessions and for scoring brain tissue fluorescence, as below.

### Water restriction schedule

An overnight water restriction schedule was imposed on study mice the day before their lickometer training commenced. This schedule aimed to motivate mice to lick fluids offered during brief-access fluid exposure training sessions. To do this, the water bottle for each home cage was removed and replaced with a marble-weighted bottle with no water, which blocked the bottle access hole on the cage top. With exceptions during sucrose sessions noted below, water restriction conditions continued through testing. While performing in training and test phases under water restriction, mice consumed their daily fluids in the lickometers. During all study phases, individual mice were given an additional 1 h access to water in their home cage post brief-access session if their daily measured body weight fell below ∼80% of their baseline weight. Food was always freely available to mice in their single-housing cages.

### Lickometer apparatus

Behavioral tests were carried out using a “Davis Rig” contact lickometer (DiLog Instruments; Med Associates). This computer-controlled device can record licking responses made by a mouse to different fluids offered on sequential, seconds-long trials while randomizing the order of fluid presentation within one test session. The short trial length and limited number of fluid trials offered during brief-access tests mitigated post-ingestive influences on licking responses, capturing ingestive/licking behavior driven by initial oral sensation (Smith, 2001; Boughter et al., 2002). In these studies, several Davis Rigs were used in parallel and in series, with mouse test order and rig assignment randomly determined daily.

Mouse licking responses to temperature-controlled water were recorded using a custom-modified Davis Rig (Li et al., 2024). This device could capture lick rates emitted to each of multiple fluid sipper tubes randomly offered to mice on discrete trials while holding the temperature of each fluid at a different set point value. During development, this device was established to hold actual fluid temperatures to within 0.2°C SD of each set point. Thermal control was achieved, in part, using feedback-operated Peltier devices that cooled or warmed independent metal blocks holding the metal shank of each proffered sipper tube. The orientation of the tubes relative to the mouse chamber mirrored the specifications of a standard Davis Rig, including positioning the fluid orifices of the sipper tubes in the access openings of a standard “lick plate.” The thermolickometer device functioned like a normal Davis Rig contact lickometer but with the capability to precisely control fluid temperatures during brief-access tests.

### Lickometer training

Overnight water-restricted mice were individually trained to receive fluids in a Davis Rig over 4 d. On training days 1 and 2, mice were allowed free access to one sipper tube filled with room temperature water for 30 min to habituate them to the apparatus. Days 3 and 4 familiarized mice with the brief-access fluid exposure procedure, offering them sipper tubes of room temperature water over 20, 10 s access trials. Once a tube was presented, mice were allowed 30 s to make a lick, which started the trial. Zero licks were recorded if no licks were made after 30 s. Intertrial intervals were 7.5 or 10 s. After completing the last day of training, mice were returned to their single-housing cage and given *ad libitum* access to water for ∼2 d, prior to beginning testing.

### CNO administration

Brief-access fluid exposure tests were performed with, and in some cases without, administration of the hM4Di agonist CNO (Sigma-Aldrich). On test days conducted under CNO influence, all mCherry and hM4Di mice in the squad received an injection (i.p.) of CNO (5 mg/kg) ∼30 min prior to the start of their brief-access session. The experimental blinding procedure described above prevented experimenters from knowing whether mice belonged to the mCherry or hM4Di group. To prepare CNO, a 5 mg vial was dissolved in 50 µl of DMSO and then transferred to sterilized saline (up to 10 ml). The CNO dose follows published recommendations for mice and was suggested to have only minimal off-target effects (Jendryka et al., 2019).

Following Cre-directed viral transduction, PB-*Calca* neurons in hM4Di mice expressed the mCherry fluorophore and hM4Di—an inhibitory DREADD engaged by CNO. Thus, administering CNO to hM4Di mice supported temporary dampening of activity in transduced PB-*Calca* neurons during brief-access tests. Receipt of CNO by mCherry mice, where transduced PB-*Calca* neurons expressed only mCherry, provided between-subjects control for nonspecific effects of this ligand (Mahler and Aston-Jones, 2018; Jendryka et al., 2019).

### Brief-access fluid exposure tests

After an approximate 2 d break with *ad libitum* access to water following training, mice returned to an overnight water restriction schedule and entered brief-access fluid exposure tests with orosensory stimuli conducted in the lickometers. All chemical stimuli were dissolved in purified water (hereafter referred to as water). Four different stimuli were tested, including temperature-controlled water (innocuous oral thermal stimuli), quinine (bitter taste), cycloheximide (bitter taste), and sucrose (appetitive taste). Brief-access test sessions with thermal and bitter stimuli performed under CNO all followed the same general format. Sucrose sessions were conducted differently and involved transitioning mice from water restriction to a water-replete (not thirst-motivated) state to accommodate the positive hedonic tone of sucrose, which is sufficient to induce licking in rodents in brief-access tests. Our between-subjects design tested mCherry control and experimental hM4Di mice in synchrony within squad, which aimed to control for potential influences of time of testing, CNO administration, and stimulus exposure. Licking responses to sucrose were also measured without CNO administration, as below.

Stimuli were specifically tested as follows:

1. Temperature-controlled water. Overnight water-restricted mice were proffered water precisely held at 15°C (mild cooling) or 35°C (innocuous warmth) over a series 5 s brief-access trials arranged in random interleaved order. Each water temperature was offered on 15 trials (30 trials total per session). Based on these parameters, mice were allowed up to 75 s of licking access to each water temperature per test session. This test was administered over 4 consecutive days, with each mouse receiving an injection of CNO ∼30 min prior to starting daily testing.
2. Quinine. Three different concentrations of the ionic bitter taste stimulus quinine-HCl (0.1, 0.3, and 1.0 mM) and water (0 mM) were proffered to overnight water-restricted mice on 10 s trials, with fluid order randomized within each of five contiguous trial blocks (20 trials total; mice were allowed up to 50 s of licking access to each concentration per session). Quinine is considered ionic as its cation and anion components are separable in water (Frank et al., 2004; Travers and Geran, 2009). Quinine solutions were presented at room temperature. This test was carried out over 4 consecutive days, with CNO administered to mice each day ∼30 min prior to the start of testing.
3. Cycloheximide. Overnight water-restricted mice were presented three different concentrations of the nonionic bitter taste stimulus cycloheximide (0.001, 0.003, and 0.01 mM) and water (0 mM) on 10 s trials, with fluid order randomized within each of five contiguous trial blocks (20 trials total; mice were allowed up to 50 s of licking access to each concentration per day). Cycloheximide solutions were offered at room temperature. These tests were conducted over 4 consecutive days under CNO, with CNO administered ∼30 min prior to testing each day.

Only 4 d with-CNO tests were performed with each bitter tastant to reduce test exposure to these stimuli and mitigate potential nonspecific effects. Water restriction, which is needed to motivate sampling of bitter-tasting fluids in brief-access settings, may cause cumulative effects on thirst motivation to lick across sequential test days (Spector and St John, 1998). Bitter taste exposure can also elevate salivary proteins that affect bitter taste sensitivity in rodents (Martin et al., 2023), with some data revealing rapid modifications of the salivary proteome after quinine taste stimulation (Quintana et al., 2009). The present between-subjects approach aimed to evenly spread latent factors and potential confounds between control and experimental conditions.

4. Sucrose. Brief-access fluid exposure tests with sucrose were performed in two phases. In Phase 1, overnight water-restricted mice were proffered four concentrations of sucrose (100, 300, 500, and 1,000 mM) and water (0 mM) at room temperature in a brief-access setting for 2 d, without CNO. Trials were 5 s long with stimulus order randomized over 8 blocks (40 trials total; mice were allowed up to 40 s of licking access to each concentration per day). Phase 1 was a training session to allow mice to learn that the sipper tubes offered sucrose—a strongly preferred taste stimulus.

During Phase 1, all mice received a simulated intraperitoneal injection prior to starting each daily brief-access session with sucrose. To do this, mice were scruffed by the neck skin behind the ears. While scruffed, mice were elevated by the experimenter's hand and a plastic syringe (without needle) was applied to abdominal skin medial to the inner thigh to mimic handling that would be received during an intraperitoneal injection. Mice were then released to their home cage. Brief-access testing began ∼30 min later. This mock injection procedure aimed to control for effects of experimenter handling and related stress on mice across sucrose sessions conducted with and without CNO.

On completion of Phase 1, mice were returned to their home cage and given unrestricted access to water. They then entered Phase 2, which began the next day. Phase 2 monitored brief-access licking responses to sucrose in water-replete (not thirst-motivated) mice with continuous access to water in their home cage. Because mice also had *ad libitum* access to food in their home cage, it was presumed that the preferred hedonic tone of sucrose, but not thirst or hunger, was the primary motivator of licking in Phase 2. CNO was administered in Phase 2. During each water-replete test session, mice were proffered four different concentrations of sucrose (100, 300, 500, and 1,000 mM) and water (0 mM) on discrete 5 s trials, with fluid order randomized within each of eight contiguous trial blocks (40 trials total; mice were allowed up to 40 s of licking access to each concentration per day). Solutions were offered at room temperature. Tests were conducted across 4 sequential days, with CNO administered ∼30 min prior to each daily session.

#### No-CNO sucrose tests

A subset of mice entered additional brief-access tests with sucrose solutions conducted as in Phase 2, but without daily administration of CNO. This no-CNO condition allowed for analysis of orosensory responses to sucrose captured with and without CNO-induced perturbation of PB-*Calca* neurons. In most cases, the 4 d no-CNO tests with sucrose began the day after the 4 d with-CNO tests completed. Mice were water-replete (not thirst-motivated) throughout the no-CNO tests. On each no-CNO test day, all mice received a mock intraperitoneal injection, as above, ∼30 min prior to starting their sucrose brief-access session. This aimed to control for potential experimenter handling effects across test sessions.

Concentration series for each taste stimulus followed published literature involving B6 mice and elicit mild or moderate to strong effects on licking behavior with increasing values (Dotson and Spector, 2004; Boughter et al., 2005). The tested water temperatures stimulate (15°C) or reduce (35°C) licking in thirst-motivated B6 mice when offered on alternate trials during a brief-access test session (Li et al., 2024).

Most mice were tested with multiple stimuli, with a break of at least 4 d separating tests with different solutions. However, no mice were tested with both cycloheximide and quinine, nor did any mouse undergo all bitter and other stimulus tests. All mice within a squad experienced the same stimuli series and test conditions. Mice received *ad libitum* access to water in their single-housing cage during break periods. Sample sizes for specific tests are detailed in the results.

### Fluorescence microscopy of brain tissue

At the conclusion of behavioral testing, mice were killed by an overdose of sodium pentobarbital (≥130 mg/kg, i.p.) and underwent transcardial perfusion with 0.9% NaCl followed by 4% paraformaldehyde and 3% sucrose dissolved in 0.1 M phosphate buffer. Brains were removed and stored in 4% paraformaldehyde and 20% sucrose dissolved in 0.1 M phosphate buffer refrigerated to ∼4°C. A sliding microtome (SM2010R, Leica) was used to cut coronal sections (20 µm) through the brain. A fluorescence microscope (Axio Scope.A1, Carl Zeiss Microscopy) and camera system (Axiocam 305 with ZEN software, Zeiss) were used by an experimenter blind to mouse group to inspect sections for mCherry (mCherry alone and hM4Di:mCherry) fluorescence labeling of neurons and processes in the PB and other brain regions. In some cases, alternate sections were Nissl stained to view brain morphology using light microscopy.

### Data analysis and statistics

The number of licks mice emitted on each brief-access trial were calculated by adding 1 to the number of interlick intervals that were >50 ms. This criterion removed potential false licks attributable to electrical/RF noise. The mean number of licks each mouse made to a stimulus per trial, across test days, was estimated by applying a 20% trimmed mean to lick counts on trials where at least one lick was recorded. Zero lick (i.e., nonsampled) trials were not counted to focus analyses on trials where mice were actively attending to the sipper tubes (St John and Boughter, 2004; Brasser et al., 2005; Ellingson et al., 2009). Mean licks per trial was estimated using a trimmed mean because stimulus lick count distributions observed for each mouse were not always normally distributed (*p* < 0.05, Jarque–Bera goodness-of-fit test). The trimmed mean is a robust measure of data location (i.e., it is resistant to outliers) that is intermediate to the median, which trims all data except the 50th percentile score, and the conventional mean, which includes all data but can be influenced by outliers and skew. The 20% trimmed mean accommodated outliers by computing average licks after dropping the lower 20% and upper 20% of lick counts considered. As a convenience, (trimmed) mean licks per trial is referred to as “licks” going forward.

Licks to the aversive bitter taste stimuli quinine and cycloheximide were also expressed as lick scores, calculated for each mouse as licks to each stimulus/concentration minus licks to water. Licks to water were obtained from analysis of water trials randomly proffered with the stimulus series during testing (i.e., the 0 mM trials). Lick scores standardized responses to aversive tastes as the difference in licking from water responding.

Licks to appetitive sucrose were analyzed using lick ratios, calculated for each mouse by dividing their licks made to each sucrose concentration by their mean primary lick rate. Mean primary lick rates were based on the mean primary interlick interval (MPI) to water observed for each mouse during the first five trials of the brief-access training days, using interlick intervals >50 and ≤160 ms. This window reflects the primary component of the interlick interval distribution, which includes most intervals/licks a mouse typically emits (Boughter et al., 2012). The reciprocal of the MPI was taken to derive the mean primary lick rate (licks/s), which was then scaled to the length of the stimulus trial under consideration (licks/5 s) to estimate the number of licks that each mouse could theoretically achieve in that timeframe with constant licking. Sucrose lick ratios that approached 1 indicated near-maximal licking; those that approached 0 reflected minimal licking. Our calculation of lick ratios is appropriate to standardize orosensory responses and gauge avidity to appetitive taste solutions like sucrose, which evokes increases in responding with rising concentration in the absence or reduction of thirst motivation (Glendinning et al., 2002; Dotson and Spector, 2004; Ellingson et al., 2009).

Latency to first lick from shutter opening was also considered for each stimulus to gauge the potential influence of olfactory/vapor cues on licking behaviors. Olfactory influences on mouse licking in brief-access assays can appear as a systematic change in latency with change in stimulus concentration (Boughter et al., 2002; Glendinning et al., 2002). For individual stimuli and mice, latency to first lick was the average (20% trimmed mean) of the latencies collected across all sampled trials.

Statistical analyses coupled inferential with estimation statistics to study the significance and magnitude of differences in licking observed between mouse DREADD groups and sexes. Parametric statistical tests, such as ANOVA, were conducted using R (R Core Team, 2022). ANOVA effect size was gauged using partial eta squared (*η*^2^). Yuen's two-sample and dependent samples trimmed mean *t* tests (WRS2 package in R; Mair and Wilcox, 2020) were applied for robust analysis of condition and group differences, including simple and main effects. Yuen's trimmed *t* retained data magnitude information (e.g., licks) as opposed to converting data points to nonparametric ranks, accommodated unequal group variances and outliers that arose in some comparisons, and provides only slightly less power than a Student's *t* test under normality (Yuen, 1974). Twenty percent trimming was used. Statistical effect size in Yuen's test was gauged by *ξ^*, where values of 0.10, 0.30, and 0.50 correspond to small, medium, and large effects, respectively (Mair and Wilcox, 2020).

Estimation analyses were carried out using Gardner–Altman plots (Gardner and Altman, 1986) custom coded in MATLAB (release 2023b, MathWorks). Here, all data points for each of two groups were displayed alongside their 20% trimmed mean and its 95% confidence interval. Next, a bootstrap approach estimated the 95% confidence interval of the mean difference between the groups (i.e., the effect size in lick response units). To do this, each data group was randomly resampled with replacement, with the number of resampled data points equal to the group sample size, and the 20% trimmed mean computed. The difference between the resampled group means was stored, with this process carried out 1,000 times. Resampled differences were then sorted in ascending order to identify the 25th (2.5%) and 975th (97.5%) entries, which defined the 95% confidence interval range for the mean difference between groups. This calculation was repeated 100 times, with the average 95% confidence interval range reported in the results. The margin of error for the mean difference was half of the average confidence interval. The expected sampling error for the mean difference was represented as a probability distribution in Gardner–Altman plots. Estimation statistics allowed for visualization and interpretation of the effect size difference between two groups in the context of effect probability, data unit of measurement, and error/group variances.

## Results

**Figure 1.**
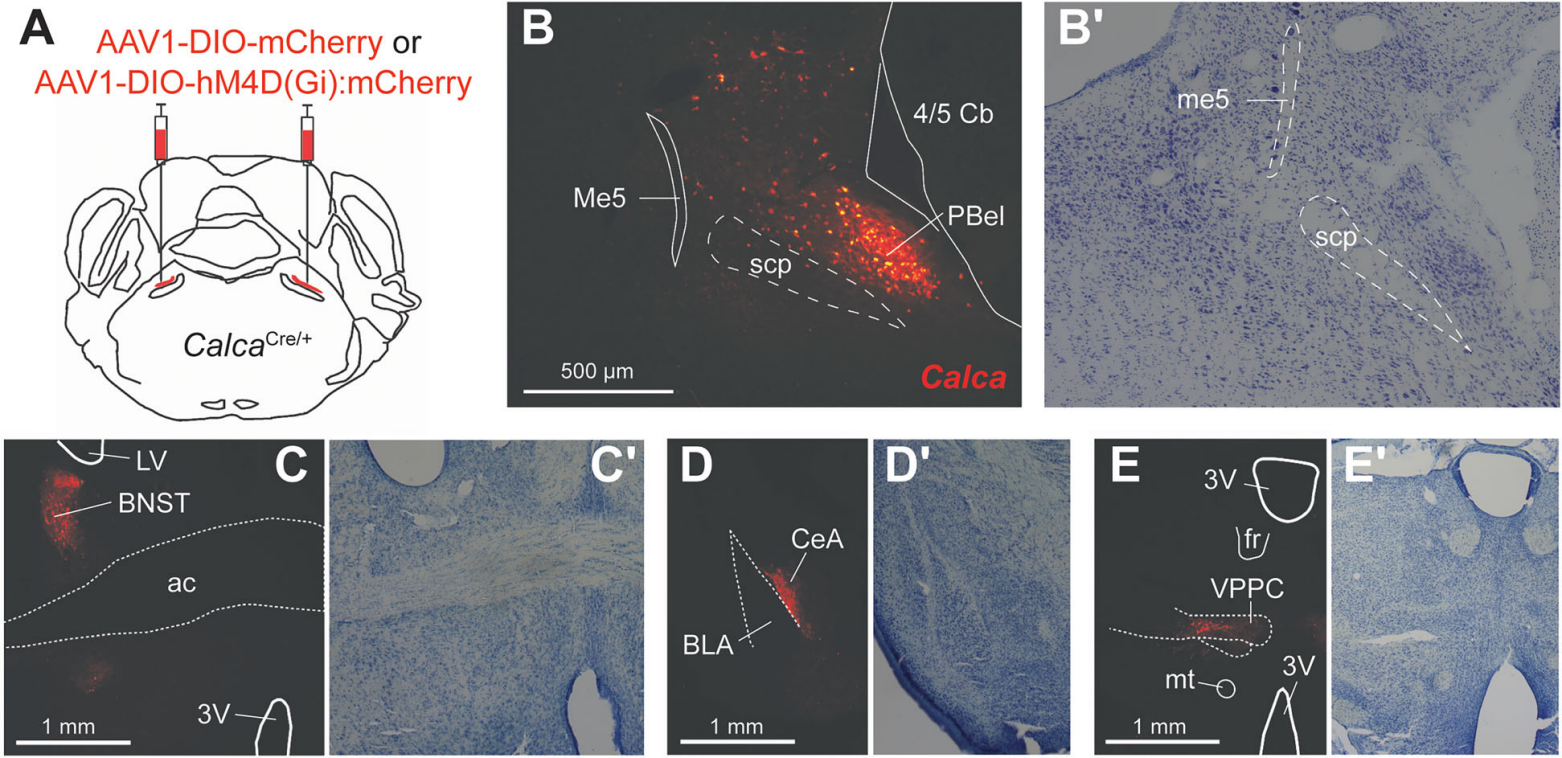
Viral transduction of PB-*Calca* neurons. ***A***, Schematic of bilateral intracranial microinjection of Cre-dependent viruses encoding either mCherry or hM4Di:mCherry to the PB area in *Calca*^Cre/+^ mice. ***B***, Microscope image of brain tissue showing Cre-dependent mCherry labeling of neurons in the external lateral parabrachial area (PBel). Nissl stain of an adjacent section is shown in ***B*′**. ***C–E***, Microscope images showing Cre-dependent mCherry labeling of anterograde processes in the bed nucleus of the stria terminalis (BNST, panel ***C***), central nucleus of the amygdala (CeA, panel ***D***), and thalamus (panel ***E***) that followed virus injection into the PB area in one *Calca*^Cre/+^ mouse. Nissl staining of adjacent sections are shown in ***C*′*–E*′**. Other abbreviations: Me5, mesencephalic trigeminal nucleus; me5, mesencephalic trigeminal tract; scp, superior cerebellar peduncle; 4/5 Cb, cerebellar lobule; LV, lateral ventricle; ac, anterior commissure; BLA, basolateral amygdaloid nucleus; 3V, third ventricle; fr, fasciculus retroflexus; VPPC, ventral posterior thalamic nucleus (parvicellular); mt, mammillothalamic tract.

Forty mCherry (19 females, 21 males) and 48 hM4Di (28 females, 20 males) mice were surgically prepared (Fig. 1*A*) and tested in these studies. We analyzed behavioral data from mice that, following postmortem microscopy of brain tissue, showed bilateral cellular expression of mCherry in the external lateral region of the PB nucleus (Fig. 1*B*), which houses a dense cluster of *Calca* neurons (Huang et al., 2021). This pattern appeared in 59 mice (67% of all examined) and was taken as evidence of successful bilateral viral transduction of PB-*Calca* neurons with fluorescence control (mCherry) or inhibitory DREADD (hM4Di:mCherry) elements. Fluorophore expression was also observed in anterograde processes within the known forebrain/limbic projection targets of PB-*Calca* cells (Shimada et al., 1985; Schwaber et al., 1988; Huang et al., 2021), including the bed nucleus of the stria terminalis, central nucleus of the amygdala, and the thalamus (Fig. 1*C–E*). Mice found to show only unilateral (*n* = 21, 24%) or no (*n* = 8, 9%) expression of mCherry in the external lateral PB area were not included in analyses.

### Viral transduction of PB-*Calca* neurons does not affect oromotor responding to water

**Figure 2.**
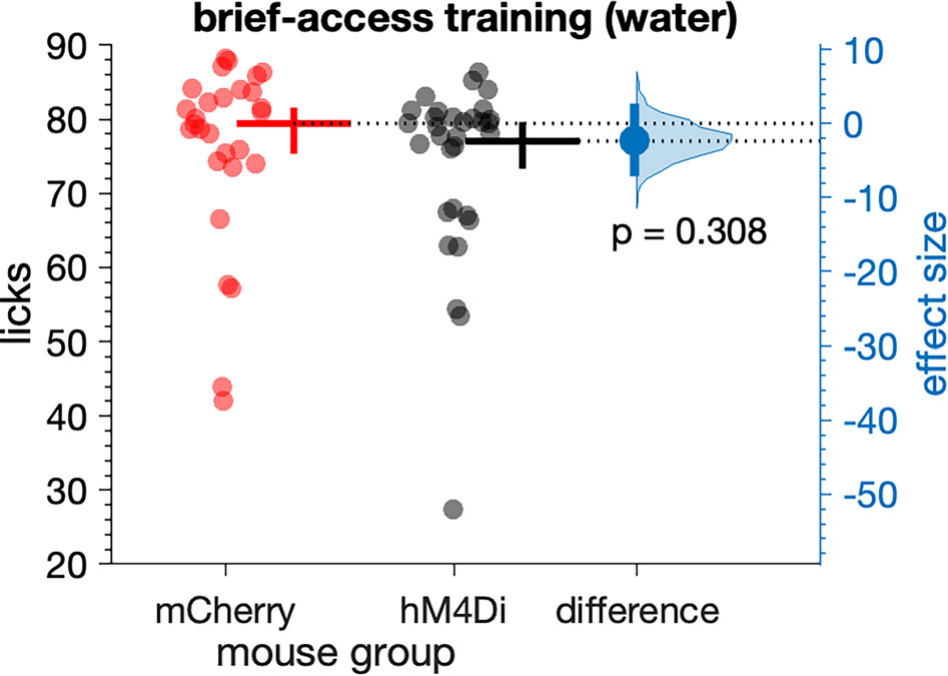
Licks emitted during brief-access training sessions with water (10 s trials) did not differ (*p* = 0.308) between mCherry (*n* = 28) and hM4Di (*n* = 31) mice (markers). The mean (20% trimmed) number of licks (horizontal bar) and its 95% confidence interval (vertical bar) is plotted to the right of each distribution. The difference (in licks) between the sample means (effect size: blue circle) is shown to the right of the plot together with its 95% confidence interval (vertical blue bar).

We examined if the expression of exogenous fluorescence and DREADD proteins in *Calca* neurons disrupted normal licking behavior. Analysis showed that licks emitted to water during the first five trials of brief-access training, without administration of CNO, did not differ between mCherry (*n* = 28) and hM4Di (*n* = 31) mice (n.s. Yuen's *t* test, *p* = 0.308; Fig. 2). Moreover, licks to water during brief-access training did not differ between female (*n* = 35) and male (*n* = 24) mice (n.s. Yuen's *t* test, *p* = 0.244).

On average, mCherry and hM4Di mice, respectively, made 79.5 and 77.1 licks during 10 s brief-access training trials with water, without CNO (Fig. 2). These lick rates approached the number of licks that B6 mice, which are a genetic background for *Calca*^Cre/+^ mice, could theoretically make with constant licking during a 10 s fluid exposure trial with room temperature water (∼81 licks), as estimated from their established peak interlick interval (∼124 ms; Boughter et al., 2007). Together, this robust licking response to water and the lack of a difference between mouse groups (Fig. 2) implies AAV-mediated transduction of PB-*Calca* neurons did not influence normal oromotor responding in the absence of CNO. These results also suggest that all mice entered lickometry tests showing similar levels of baseline responding.

### CNO does not affect preferences for innocuous oral temperatures in *Calca*;hM4Di mice

Our prior data show that when given a choice, thirst-motivated mice prefer to lick water at a mild cool (15°C) rather than innocuous warm (35°C) temperature in brief-access tests conducted with temperature-controlled fluids (Li et al., 2024). Here, we examined if chemogenetic dampening of PB-*Calca* neurons would affect this behavior to gauge their role in preferences for innocuous orosensory stimuli.

**Figure 3.**
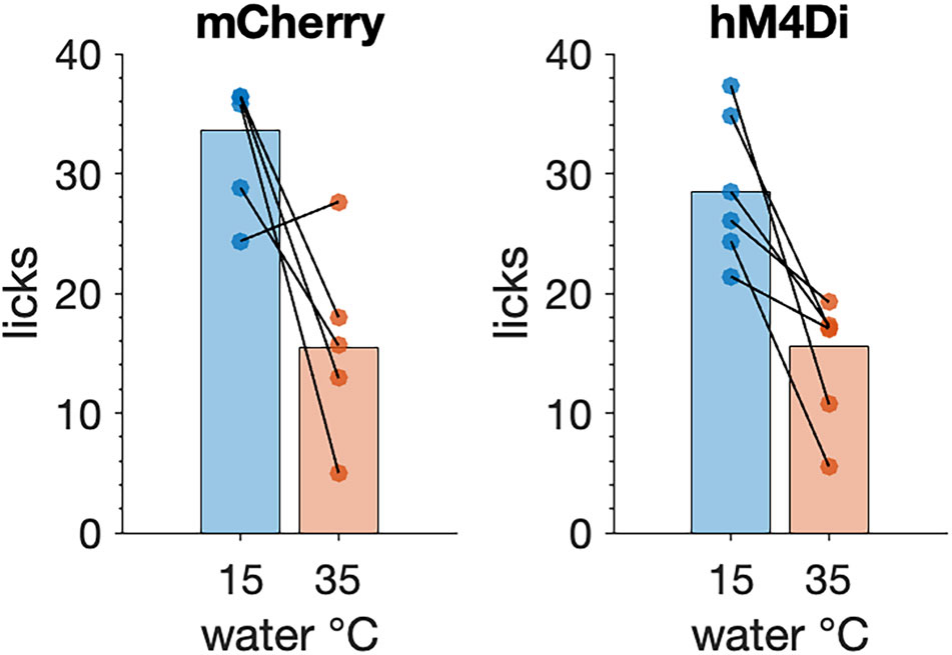
Both mCherry (*n* = 5) and hM4Di (*n* = 6) mice licked more to 15°C (mild cool) than 35°C (innocuous warm) water during brief-access fluid exposure tests conducted under CNO (*p* = 0.00098). Trials were 5 s long. This effect was similar between mouse groups (*p* = 0.734). Lines connect responses (markers) made by one mouse. Bars are 20% trimmed means.

A two-way ANOVA applied to data from 5 mCherry (3 females, 2 males) and 6 hM4Di (2 females, 4 males) mice undergoing water restriction revealed that 15°C water evoked more licks than 35°C water under CNO (main effect of temperature: *F*_(1,9)_ = 22.98, *p* = 0.00098, partial *η*^2^ = 0.719; Fig. 3). This preference for mild cool over warm water agrees with prior results (Li et al., 2024) and did not differ between mCherry and hM4Di mice (n.s. mouse group × temperature interaction, *p* = 0.734). Thus, PB-*Calca* neurons do not affect licking preferences toward innocuous orosensory cues, at least in the context of the thermal stimuli tested here.

### CNO variably reduces bitter taste avoidance in *Calca*;hM4Di mice

#### Quinine

**Figure 4.**
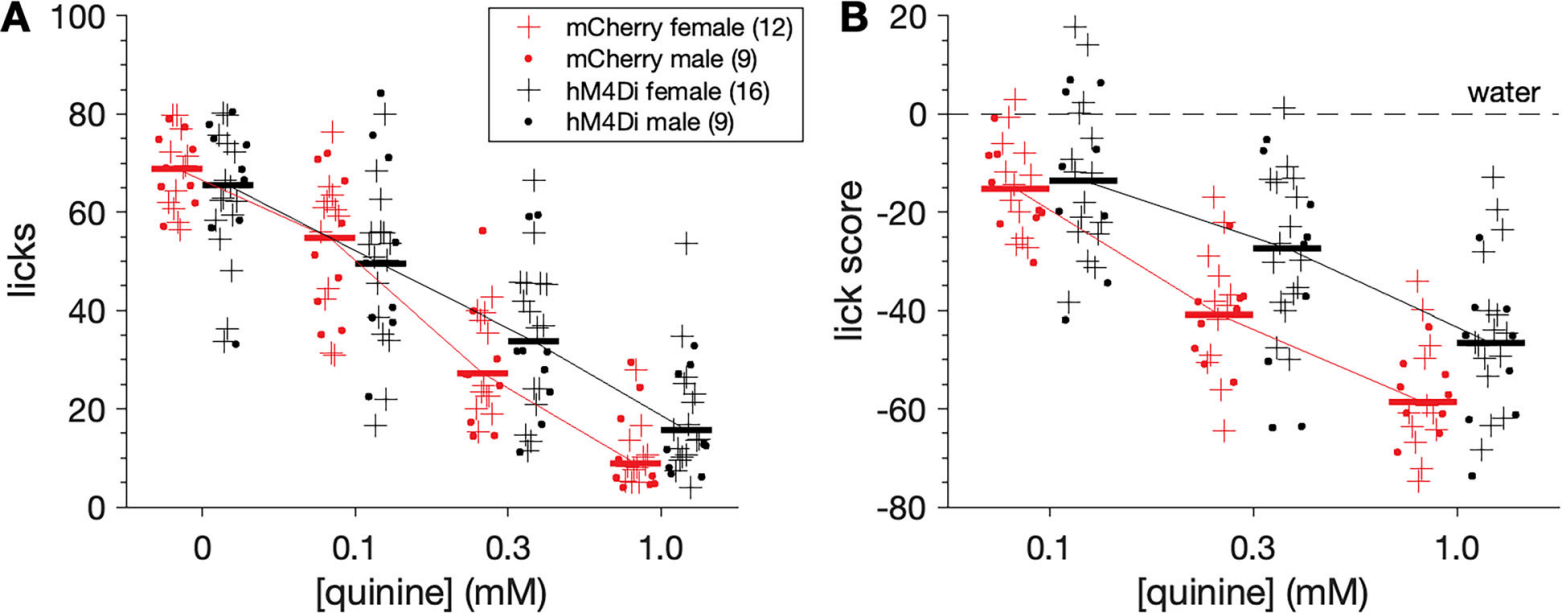
hM4Di mice show reduced orosensory avoidance of quinine while under CNO. ***A***, Concentration-response functions showing the number of licks to water (0 mM) and quinine solutions (0.1–1.0 mM) for all mice (markers) in both mouse groups and sexes (legend). Trials were 10 s long. ***B***, Quinine concentration-response functions replotted using lick scores, where points represent the number of stimulus licks minus water licks for all mice. Horizontal bars in both panels are 20% trimmed means. Factorial analyses revealed that stimulus concentration and mouse line interacted to influence licks (*p* = 0.0084) and lick scores (*p* = 0.044) to quinine.

Orosensory responses to the ionic bitter taste stimulus quinine were compared between 21 mCherry (12 females, 9 males) and 25 hM4Di (16 females, 9 males) water-restricted mice. While under CNO, mCherry and hM4Di mice made similar licks to water (0 mM) offered with quinine solutions during brief-access tests (n.s. independent samples *t* test, *p* = 0.104; Fig. 4*A*). A three-way ANOVA revealed that adulterating water with quinine decreased licking in both mouse groups as quinine concentration increased (main effect of quinine concentration, *F*_(3,126)_ = 212, *p* < 0.001, partial *η*^2^ = 0.835; Fig. 4*A*). Yet the degree of this effect differed between mCherry and hM4Di mice (mouse group × quinine concentration interaction, *F*_(3,126)_ = 4.08, *p* = 0.0084, partial *η*^2^ = 0.089), with hM4Di mice appearing to show, on average, less of a reduction in responding (i.e., greater licks to quinine) while under CNO.

**Figure 5.**
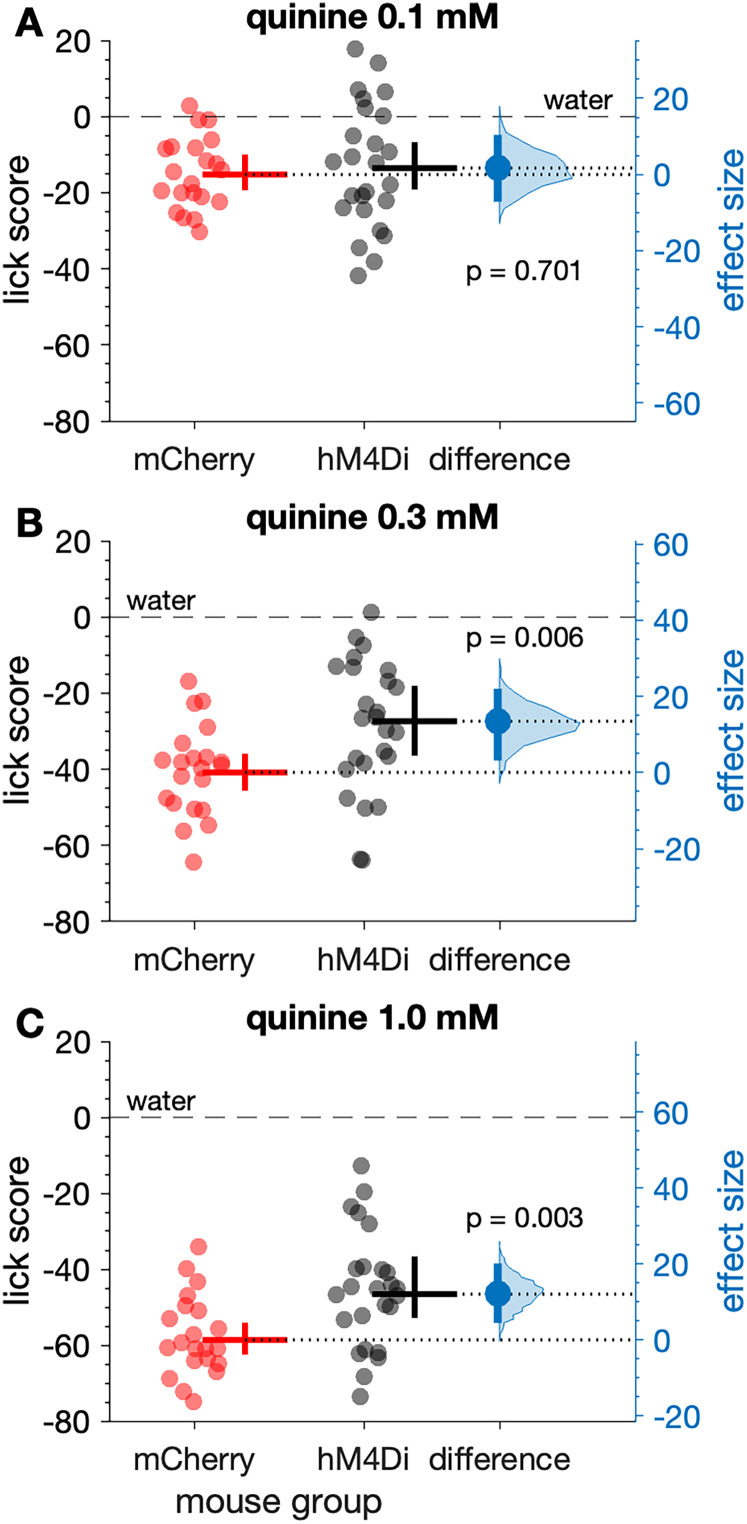
Lick scores to elevated concentrations of quinine were larger in hM4Di (*n* = 25) compared with mCherry (*n* = 21) mice (markers), all receiving CNO. Lick scores to 0.1 mM quinine did not differ between mCherry and hM4Di mice (***A***, *p* = 0.701). In contrast, lick scores to 0.3 mM (***B***, *p* = 0.006) and 1.0 mM (***C***, *p* = 0.003) quinine were higher in hM4Di mice under CNO. Trials were 10 s long. For each panel, the mean (20% trimmed) number of licks (horizontal bar) and its 95% confidence interval (vertical bar) is plotted to the right of each distribution. The difference between the sample means (effect size: blue circle) is shown to the right of the plot together with its 95% confidence interval (vertical blue bar).

Analysis of lick scores, which standardized responses as reductions in licks from water licking, revealed that lick differences to quinine between mCherry and hM4Di mice were conditioned on stimulus concentration (three-way ANOVA: mouse group × quinine concentration interaction, *F*_(2,84)_ = 3.26, *p* = 0.044, partial *η*^2^ = 0.072; Fig. 4*B*). Robust simple effects tests found that while under CNO, lick scores to 0.1 mM quinine did not differ between mouse groups (n.s. Yuen's *t* test, *p* = 0.701; Fig. 5*A*). However, 0.3 mM quinine evoked higher lick scores in hM4Di mice (Yuen's *t* test, *t*_(23.6)_ = 3.01, *p* = 0.006, *ξ^* = 0.55). On average, hM4Di mice emitted ∼14 more licks to 0.3 mM quinine (a 33% increase) than mCherry mice during 10 s trials, with a 95% confidence interval for this difference of 3–22 more licks (Fig. 5*B*). The margin of error for this increase was ∼9 licks, which was lower than the effect size of 14 licks.

Moreover, lick scores to 1.0 mM quinine were higher in hM4Di compared with mCherry mice (Yuen's *t* test, *t*_(25.2)_ = 3.29, *p* = 0.003, *ξ^* = 0.60), with hM4Di mice making ∼12 more mean licks (a 21% increase) in 10 s trials compared with mCherry controls (Fig. 5*C*). The 95% confidence interval for this increase was 4–20 more licks (margin of error = 8 licks).

Although some of the largest increases in responding to quinine observed in hM4Di mice emerged in females (Fig. 4*A*,*B*), sex did not significantly interact with mouse group and quinine concentration to influence quinine lick scores under CNO (n.s. three-way interaction, *p* = 0.582; n.s. sex × mouse group interaction, *p* = 0.661). Finally, latency to first lick was not influenced by mouse group or quinine concentration (n.s. mouse group × concentration interaction, *p* = 0.704; n.s. effect of mouse group, *p* = 0.326; n.s. effect of concentration, *p* = 0.119), which suggested olfactory/vapor cues did not affect responses to quinine.

#### Cycloheximide

**Figure 6.**
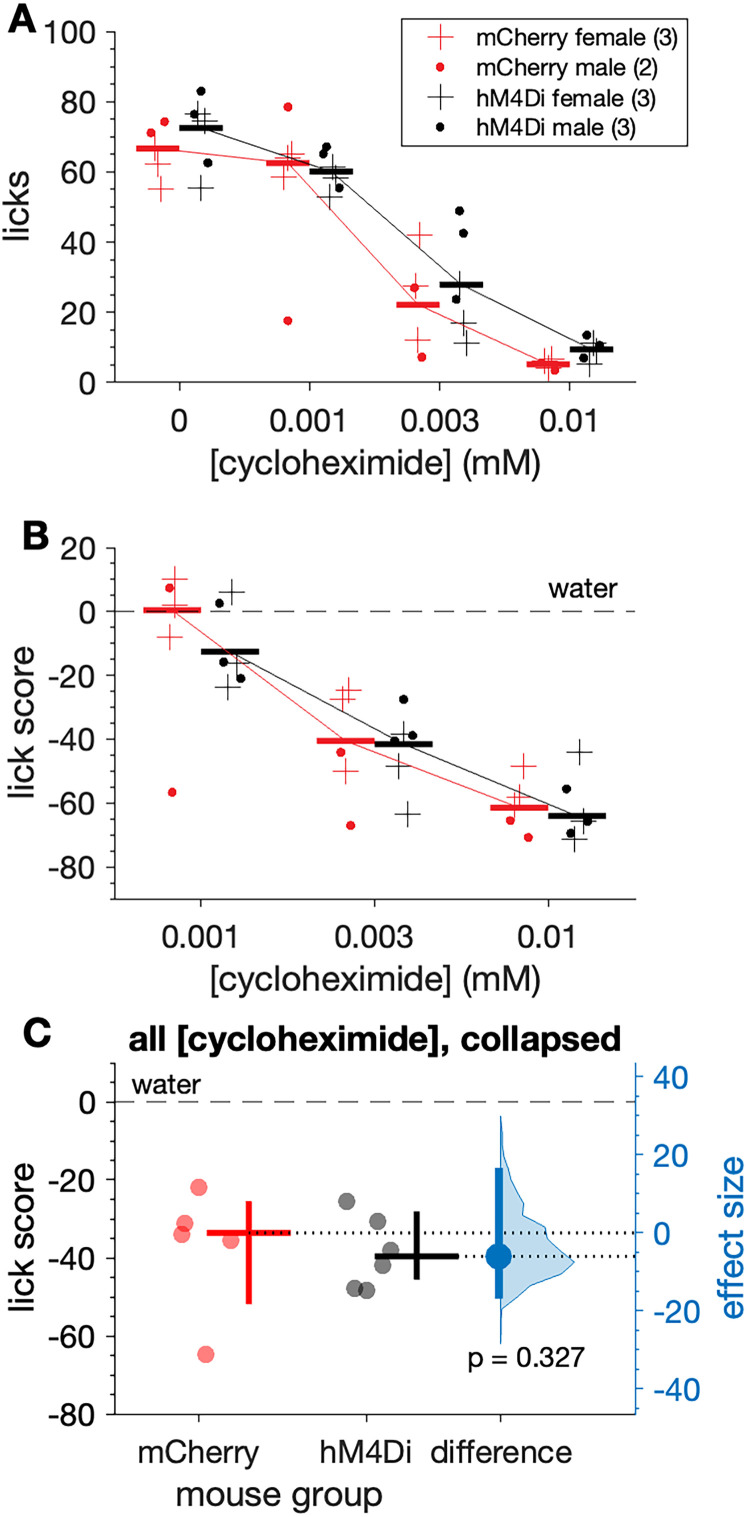
Orosensory avoidance of cycloheximide did not differ between hM4Di and mCherry mice under CNO. Plots show concentration-response functions for cycloheximide licks (***A***) and lick scores (***B***) by hM4Di (*n* = 6) and mCherry (*n* = 5) mice (markers) administered CNO. Trials were 10 s long. Horizontal bars are 20% trimmed means. ***C***, Lick scores to cycloheximide collapsed across concentrations did not differ (*p* = 0.327) between mouse groups. The mean (20% trimmed) number of licks (horizontal bar) and its 95% confidence interval (vertical bar) is plotted to the right of each distribution. The difference between the sample means (effect size: blue circle) is shown to the right of the plot along with its 95% confidence interval (vertical blue bar).

Orosensory responses to the bitter taste stimulus cycloheximide were examined in a different cohort of 5 mCherry (3 females, 2 males) and 6 hM4Di (3 females, 3 males) water-restricted mice. Under CNO, mCherry and hM4Di mice showed similar licks to water offered with cycloheximide solutions during tests (n.s. independent samples *t* test, *p* = 0.330; Fig. 6*A*). Both mouse groups showed concentration-dependent reductions in licks (two-way ANOVA: main effect of concentration, *F*_(3,27)_ = 74.21, *p* < 0.001, partial *η*^2^ = 0.892; Fig. 6*A*) and lick scores (two-way ANOVA: main effect of concentration, *F*_(2,18)_ = 61.85, *p* < 0.001, partial *η*^2^ = 0.873; Fig. 6*B*) to cycloheximide. Yet unlike quinine, these reductions were similar between groups (n.s. mouse group × concentration interaction on lick scores, *p* = 0.974). Relatedly, mean lick scores to cycloheximide collapsed across concentration did not differ between hM4Di and mCherry mice under CNO (n.s. Yuen's *t* test, *p* = 0.327; Fig. 6*C*). Underpowered sample sizes precluded analyses of cycloheximide data by sex, albeit no observable differences between females and males appeared in plotted data (Fig. 6*A*,*B*).

Altogether, these results indicate that CNO variably suppressed orosensory avoidance of bitter taste stimuli in mice where the inhibitory DREADD hM4Di was expressed in PB-*Calca* neurons. In these (hM4Di) mice, CNO significantly increased licking to quinine solutions with some variance in this increase noted across animals. Based on this variance, a range of only marginal to larger increases in responding to quinine under CNO was compatible with our data. Unlike quinine, orosensory responses to cycloheximide were not significantly influenced by CNO. While there are some limitations to comparing the present quinine and cycloheximide results, the different effects of CNO observed between these stimuli associate with known functional differences between them, as discussed below.

### CNO causes sex-dependent reductions in sucrose taste preference in *Calca*;hM4Di mice

**Figure 7.**
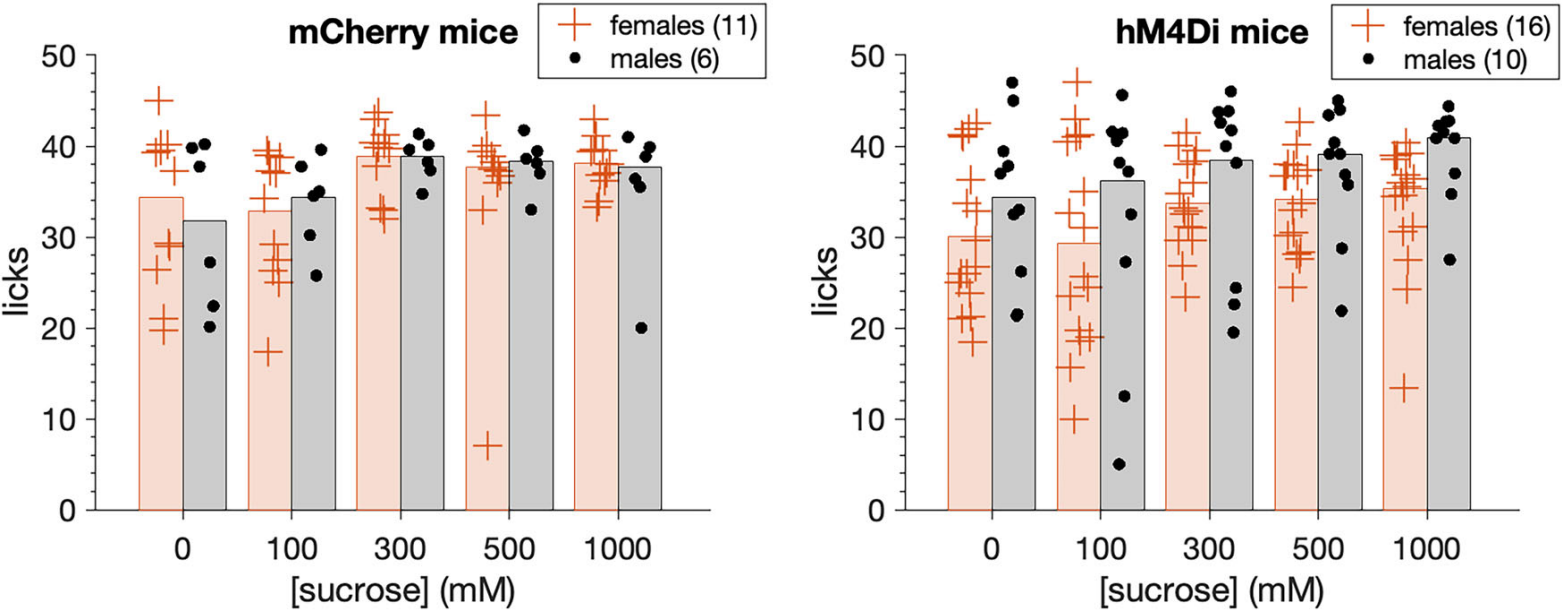
Water-restricted mCherry (*n* = 17) and hM4Di (*n* = 26) mice (markers) increased (*p* = 0.00013) their licks emitted to sucrose solutions at elevated stimulus concentrations during pre-CNO sucrose training sessions. These elevations were similar between mouse groups (*p* = 0.794) and sexes (*p* = 0.620). Trials were 5 s long. Bars are 20% trimmed means.

We analyzed orosensory responses to sucrose solutions collected from 17 mCherry (11 females, 6 males) and 26 hM4Di (16 females, 10 males) mice. Both mCherry and hM4Di mice emitted increased licks to sucrose at elevated stimulus concentrations during the Phase 1 sucrose training sessions (three-way ANOVA: main effect of concentration, *F*_(4,156)_ = 6.14, *p* = 0.00013, partial *η*^2^ = 0.136; Fig. 7). Phase 1 was conducted under water restriction conditions and aimed to orient mice to the availability of sucrose in the sipper tubes, prior to CNO tests. The increases in licking to elevated sucrose were similar between mCherry and hM4Di mice (n.s. mouse group × sucrose concentration interaction, *p* = 0.794) and between sexes (n.s. sex × mouse group × sucrose concentration interaction, *p* = 0.620; n.s. sex × mouse group interaction, *p* = 0.278), implying mice subsequently entered sucrose testing displaying similar levels of responding.

**Figure 8.**
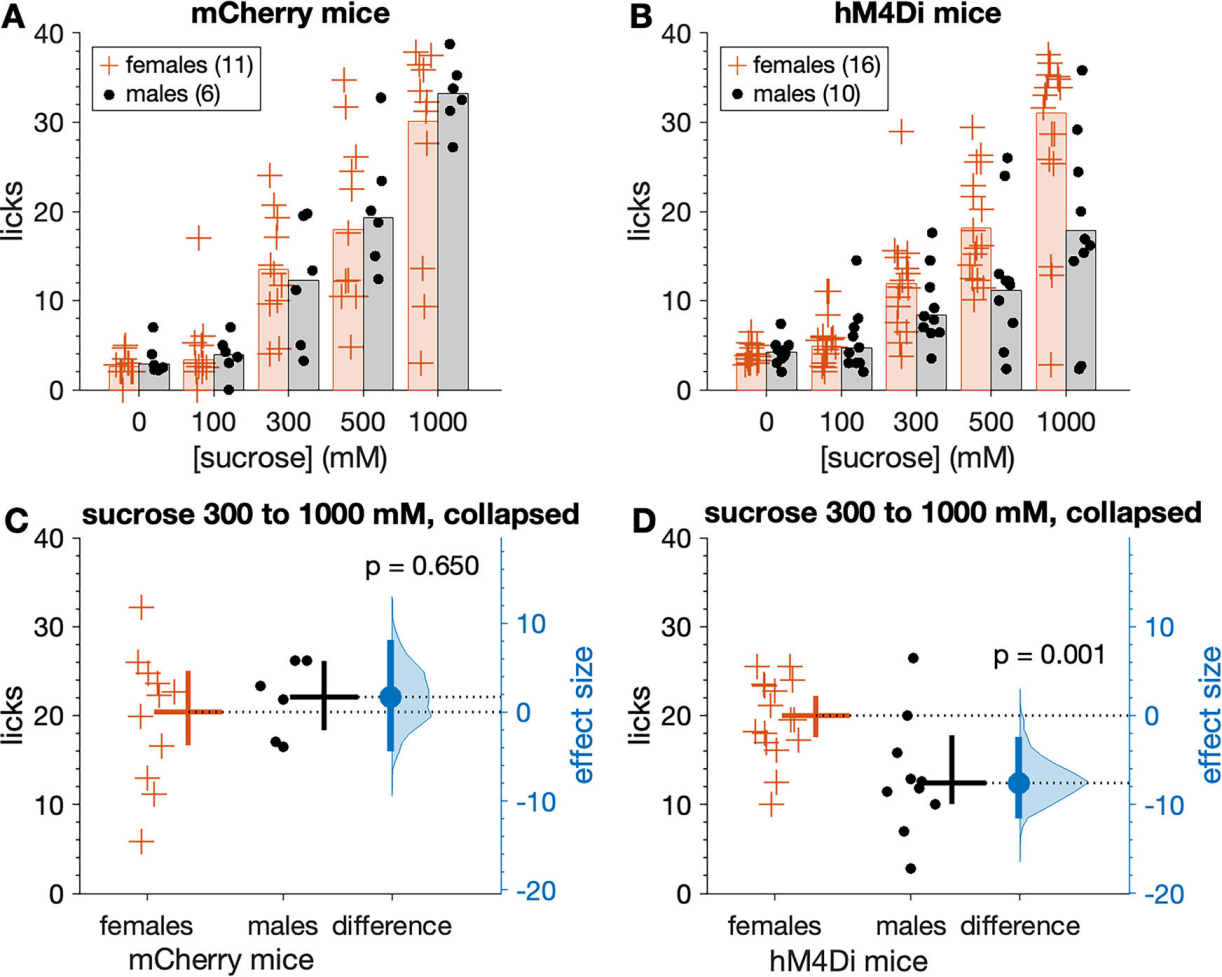
Male hM4Di mice show reduced licks to sucrose under CNO when water replete (not thirst-motivated). Top row shows the number of licks emitted across the sucrose series for mCherry (***A***) and hM4Di (***B***) mice (markers). Trials were 5 s long. Bars are 20% trimmed means. When collapsed across the three highest (most salient) concentrations, mean licks to sucrose did not differ between female and male mCherry mice (***C***, *p* = 0.650). However, male hM4Di mice made fewer licks to sucrose than female hM4Di mice (***D***, *p* = 0.001). Female hM4Di mice responded similarly to mCherry control mice (*p* = 0.496; see text for additional details). In panels ***C*** and ***D***, the mean (20% trimmed) number of licks (horizontal bar) and its 95% confidence interval (vertical bar) is plotted to the right of each distribution. The difference (in licks) between the sample means (effect size: blue circle) is shown to the right of the plot together with its 95% confidence interval (vertical blue bar).

Following sucrose training, mCherry and hM4Di mice began Phase 2 brief-access tests with the sucrose series while water replete (not thirst-motivated) and after receiving CNO. A three-way ANOVA revealed that in the absence of thirst drive, both mouse groups showed concentration-dependent increases in licks to sucrose when tested under CNO (main effect of concentration, *F*_(3,117)_ = 77.72, *p* < 0.0001, partial *η*^2^ = 0.666; Fig. 8*A*,*B*). However, the magnitude of these increases differed between mCherry and hM4Di mice in a manner that was conditioned on sex (sex × mouse group × sucrose concentration interaction, *F*_(3,117)_ = 3.15, *p* = 0.027, partial *η*^2^ = 0.075). Inspection of plotted data revealed that orosensory responses to sucrose were markedly and selectively impaired in male hM4Di mice, which displayed, on average, fewer licks to elevated sucrose concentrations than female hM4Di, and all mCherry, mice (Fig. 8*A*,*B*).

When data were collapsed across the three highest (most salient) sucrose concentrations tested (300, 500, and 1,000 mM), male hM4Di mice made significantly fewer licks to sucrose than female hM4Di mice, all administered CNO (Yuen's *t* test, *t*_(11.8)_ = 4.31, *p* = 0.001, *ξ^* = 0.78). Specifically, male hM4Di mice emitted, on average, approximately eight less licks to sucrose (a 38% decrease) during 5 s exposure trials compared with hM4Di females (Fig. 8*D*). The 95% confidence interval for this decrease was ∼2–12 less licks, with a margin of error of five fewer licks. In contrast, salient concentrations of sucrose evoked similar numbers of licks in female and male mCherry mice administered CNO (n.s. Yuen's *t* test, *p* = 0.650; Fig. 8*C*), which both licked sucrose at the same rate as female hM4Di mice (n.s. Yuen's *t* test, *p* = 0.496).

**Figure 9.**
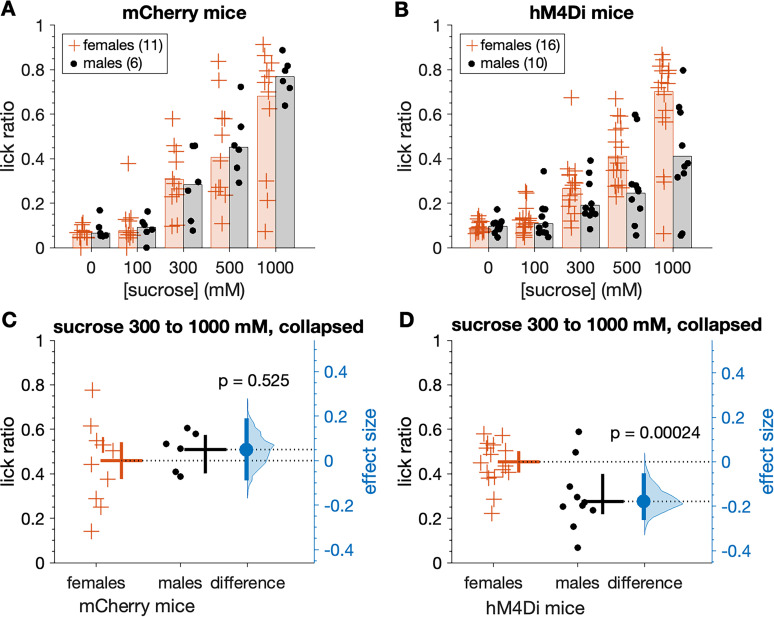
Male hM4Di mice show reduced lick ratios to sucrose under CNO when water replete. ***A***, Sucrose lick ratios for mCherry mice (markers). Trials were 5 s long. Bars are 20% trimmed means. ***B***, same as ***A*** but for hM4Di mice. When collapsed across the three highest (most salient) concentrations, mean lick ratios to sucrose did not differ between female and male mCherry mice (***C***, *p* = 0.525). In contrast, mean sucrose lick ratios were reduced in male compared with female hM4Di mice (***D***, *p* = 0.00024). See text for further details. In panels ***C*** and ***D***, the mean (20% trimmed) lick ratio (horizontal bar) and its 95% confidence interval (vertical bar) is plotted to the right of each distribution. The difference between the sample means (effect size: blue circle) is shown to the right of the plot together with its 95% confidence interval (vertical blue bar).

The same trend emerged when brief-access licking responses were standardized for each mouse using lick ratios, where their lick count to each sucrose concentration was divided by an estimate of their maximal potential licking rate. Lick ratios to sucrose captured under CNO increased with elevations in sucrose concentration in a mouse group- and sex-dependent manner, with male hM4Di mice displaying a unique reduction in lick ratio responses compared with the other mouse groups (three-way ANOVA, sex × mouse group × sucrose concentration interaction, *F*_(3,117)_ = 3.29, *p* = 0.023, partial *η*^2^ = 0.078; Fig. 9*A*,*B*). Specifically, lick ratios collapsed across 300, 500, and 1,000 mM sucrose were significantly lower, by 39%, in male hM4Di compared with female hM4Di mice under CNO (Yuen's *t* test, *t*_(12.6)_ = 5.06, *p* = 0.00024, *ξ^* = 0.75; Fig. 9*D*). The 95% confidence interval for this reduction ranged from 11% to 58% lower (margin of error = 23%). On the other hand, lick ratios collapsed across 300, 500, and 1,000 mM sucrose did not differ between female and male mCherry mice (n.s. Yuen's *t* test, *p* = 0.525; Fig. 9*C*), nor did they differ between all mCherry and female hM4Di mice (n.s. Yuen's *t* test, *p* = 0.432). Finally, male hM4Di mice displayed lick ratios to 300, 500, and 1,000 mM sucrose (collapsed) that were significantly lower compared with all mCherry control mice (Yuen's *t* test, *t*_(14.8)_ = 5.17, *p* = 0.00012, *ξ^* = 0.76). Thus, male hM4Di mice selectively made reduced licks for sucrose under CNO.

The lick ratio standardization used here is appropriate to gauge orosensory avidity to appetitive taste stimuli that stimulate licking, like sucrose (Glendinning et al., 2002; Dotson and Spector, 2004; Ellingson et al., 2009). Notably, many mCherry and female hM4Di mice displayed lick ratios that approached 1 (maximal licking) for the highest sucrose concentrations tested, albeit most male hM4Di mice showed markedly reduced ratio responding (Fig. 9*A*,*B*).

Finally, latency to first lick to sucrose solutions did not differ across concentrations (three-way ANOVA, n.s. effect of sucrose concentration, *p* = 0.834) and was not influenced by mouse group (n.s. mouse group × concentration interaction, *p* = 0.656; n.s. effect of group, *p* = 0.415) or sex (n.s. sex × mouse group × concentration interaction, *p* = 0.999). Thus, olfactory/vapor cues did not appear to impact orobehavioral responses to sucrose.

Altogether, these and the above data imply that CNO suppressed hedonic licking of sucrose selectively in male hM4Di mice, where the inhibitory DREADD hM4Di was expressed in PB-*Calca* neurons. This result suggests that *Calca* neuron participation in appetitive taste is related to sex.

**Figure 10.**
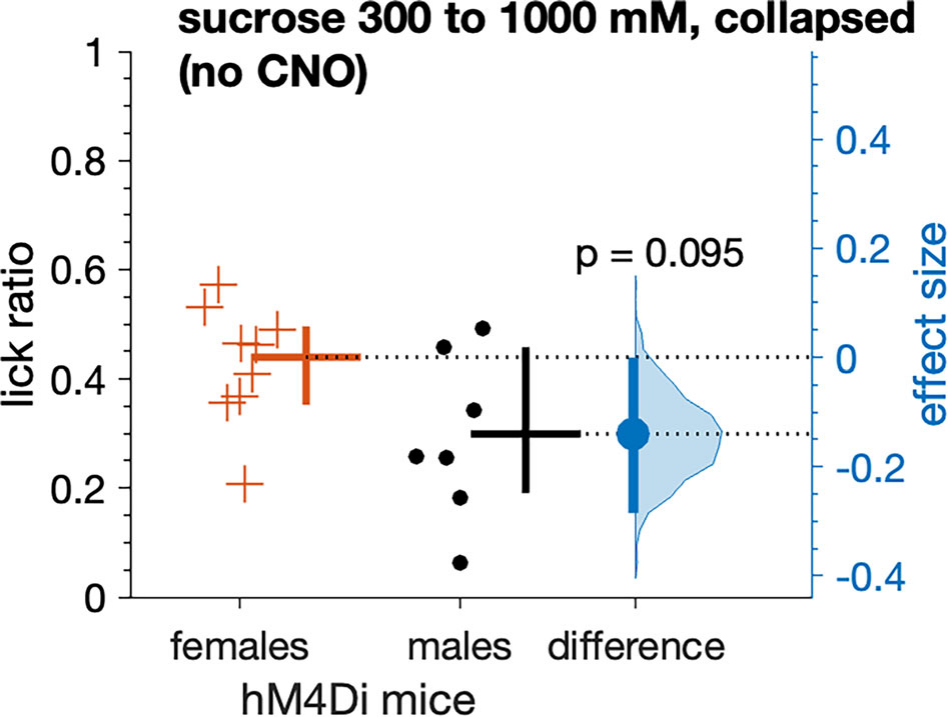
Without CNO, average lick ratios to sucrose, collapsed across the three highest concentrations, did not differ between female and male hM4Di mice (*p* = 0.095). However, some individual males showed reduced responding. Trials were 5 s long. The mean (20% trimmed) lick ratio (horizontal bar) and its 95% confidence interval (vertical bar) is plotted to the right of each distribution. The difference between the sample means (effect size: blue circle) is shown to the right of the plot together with its 95% confidence interval (vertical blue bar).

A subset of the female (*n* = 9) and male (*n* = 7) hM4Di mice examined for sucrose preferences were also tested in a brief-access setting with sucrose performed without daily administration of CNO. In these tests, no significant difference in mean responding between sexes was found (n.s. Yuen's *t* test, *p* = 0.095; Fig. 10). Nevertheless, inspection of the plotted data and sex difference confidence interval for the no-CNO tests implied that sucrose responses by males did not fully match female levels, with some reduction in male responding compatible with our data (Fig. 10).

**Figure 11.**
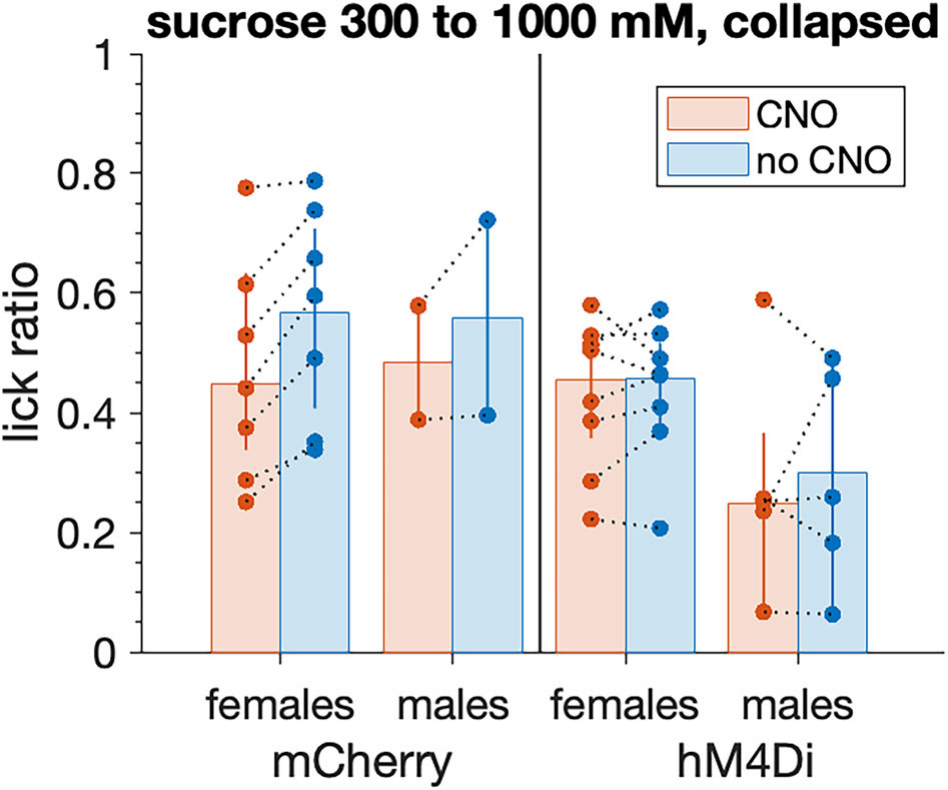
Lick ratios to sucrose during sequential with- and no-CNO tests. Responses by individual mice (circles) are collapsed across the three highest sucrose concentrations and connected by dotted lines. Trials were 5 s long. Sucrose lick ratios emitted by male hM4Di mice (*n* = 5) did not differ across sequential with- and no-CNO sessions (n.s. Yuen's *t* test for dependent samples, *p* = 0.696). The same result emerged for female hM4Di mice (*n* = 8, *p* = 0.545), which showed elevated sucrose lick ratios compared with hM4Di males. Similarly elevated sucrose lick ratios appeared for female (*n* = 7) and male (*n* = 2) mCherry mice. Female mCherry mice showed significantly higher lick ratio responding to sucrose during the no-CNO than with-CNO tests (*p* = 0.003). Bars are 20% trimmed means, with vertical lines giving their 95% confidence intervals.

Moreover, the mean response to sucrose by male hM4Di mice did not differ between the with- and no-CNO conditions when the latter was tested last (n.s. Yuen's *t* test for dependent samples, *p* = 0.696; Fig. 11). Thus, overall, hM4Di males did not increase their responding to sucrose after CNO administration was discontinued. This result may reflect carryforward of reduced responding induced by CNO. Unlike hM4Di mice, mCherry mice increased their licking of sucrose during the no-CNO tests (females: Yuen's *t* test for dependent samples, *t*_(4)_ = −6.33, *p* = 0.003, *ξ^* = 0.33; males: insufficient data for analysis). These effects are further discussed below.

## Discussion

The present study provides evidence that PB-*Calca* neurons participate in mouse orosensory responses to hedonically diverse taste stimuli. Data arose from brief-access fluid exposure tests blindly conducted on hM4Di mice, which were prepared for hM4Di-mediated dampening of activity in PB-*Calca* neurons, and mCherry control mice, which expressed only a fluorophore in PB-*Calca* cells. Before daily tests, the hM4Di ligand CNO was administered to both hM4Di and mCherry mice, with the latter group intended to control for potential nonspecific CNO effects (Mahler and Aston-Jones, 2018; Jendryka et al., 2019). Under these experimental conditions, hM4Di mice showed increased licking acceptance of the bitter taste stimulus quinine compared with mCherry mice, with observed variability in the magnitude of this effect. Furthermore, male hM4Di mice made fewer licks for the appetitive sugar (sweet) sucrose compared with control and female hM4Di mice. Female hM4Di mice showed sucrose preferences like those of mCherry control mice. During daily test sessions, mice were able to lick each taste stimulus concentration for up to a maximum of 40 s (sucrose) or 50 s (bitter stimuli). Thus, the noted effects arose during brief encounters with these stimuli, reflecting sensory-guided responses.

### A role for PB-*Calca* neurons in sweet taste

A dual influence on aversive (quinine) and preferred (sucrose) tastes may appear perplexing in the context that PB-*Calca* neurons were established to have roles in protective responses to unfavorable and noxious conditions (Carter et al., 2013; Campos et al., 2018; Kang et al., 2022). Selective toxin-induced inactivation of PB-*Calca* neurons was also reported to have no effect on mouse licking preferences to the noncaloric sweetener saccharin (Jarvie et al., 2021). However, recent data have implied these neurons display a more diverse response repertoire that includes activation to appetitive stimuli (Kim et al., 2024b). Notably, PB-*Calca* neurons appear to respond to preferred and aversive stimuli by changes in response frequency and use of frequency-modulated neurotransmission. In mice, drinking sucrose (appetitive) and tail pinch (aversive) caused low and high rates of spiking in PB-*Calca* neurons that respectively associated with preferential release of glutamate or neuropeptides, which may contribute different component features to sensations (Kim et al., 2024a). The present results suggest that participation of PB-*Calca* neurons in appetitive taste-guided behavior is conditioned on sex, with normal functioning in PB-*Calca* cells evidenced to be required for male, but not female, mice to express levels of licking preference to sucrose observed in control animals. Prior studies that examined how PB-*Calca* neurons may affect mouse fluid licking that was associated with taste did not address or report sex effects.

There are some features of these data to consider further. A subset of mice was tested with sucrose in brief-access tests conducted without CNO administration. Inferential statistics revealed that in the absence of CNO, mean responses to sucrose did not significantly differ between female and male hM4Di mice (Fig. 10). This implied the reduction in sucrose responding by males during the with-CNO tests (Figs. 8*D*, 9*D*) was due to DREADD influence on PB-*Calca* cells. Yet estimation statistics and plots suggested that some hM4Di males did respond less to sucrose compared with other mouse groups during the no-CNO tests, with most of these tests conducted after the with-CNO condition (Fig. 11).

As is common in behavioral studies of this type, the present design was unable to account for all extraneous factors that may have potentially affected mouse orosensory preference responses. Because of this, we cannot fully rule out that a blunted sucrose response by male hM4Di animals was influenced by a latent variable. Nonetheless, all mouse groups and sexes showed similar, robust levels of licking to the sucrose series during training sessions that preceded the onset of the sucrose tests (Fig. 7). This implies all mice began these tests displaying similar levels of baseline activity. Moreover, mCherry mice administered CNO showed concentration-dependent licking responses to sucrose, and quinine, typical of those reported in brief-access tests conducted with common inbred B6 mice under water-replete and water restriction conditions (Dotson and Spector, 2004; Ellingson et al., 2009), supporting use of these animals as controls for unspecific effects.

We observed that mCherry mice increased their licking of sucrose during the no-CNO tests that followed the with-CNO test days (Fig. 11). While possibly reflecting a building preference for sucrose in control animals, this result may also associate with a potential confound of the no-CNO condition. During the no-CNO tests, mice were handled to control for stress associated with the CNO injection procedure, but they did not receive a fluid injection as during the with-CNO assay. This could have resulted in a change in hydration status between conditions that impacted licking behavior. Nonetheless, mice were not water restricted during all with- and no-CNO test sessions with sucrose, suggesting thirst would not play a major role in licking during the present sucrose tests. Moreover, female and male hM4Di mice did not show increased licks to sucrose during the no-CNO tests, with hM4Di males displaying lingering attenuated sucrose preference without CNO. This finding implies that even with potential influences of injection hydration removed, hM4Di males did not recover control-like preferences for sucrose.

Speculatively, a continued reduced avidity for sucrose by hM4Di males could arise from carryforward or learning phenomena that developed while these mice consumed sucrose during an altered PB network state under CNO and potential differences in PB and downstream circuits between sexes. Prior studies have implicated PB-*Calca* neurons with roles in ingestive learning (Carter et al., 2015; Chen et al., 2018). PB neurons expressing *Calca*/CGRP project to the central nucleus of the amygdala (Fig. 1*D*; Shimada et al., 1985; Schwaber et al., 1988; Huang et al., 2021), which has been implicated with roles in taste palatability processing (Touzani et al., 1997; Lundy, 2008; Sadacca et al., 2012; Bartonjo et al., 2022). While we are not aware of functional data that address sex differences in sucrose taste responses by PB-*Calca* neurons, CGRP-related signaling in the central amygdaloid nucleus is associated with sex differences in affective function related to pain (Presto and Neugebauer, 2022). What is more, taste-active PB neurons show differences in gustatory responses to sucrose between female and male rats that appear insensitive to changes in ovarian hormones in adulthood, suggesting there are organizational differences in PB gustatory circuits between sexes (Di Lorenzo and Monroe, 1989, 1990). Future neurophysiological studies may bolster understanding of *Calca* neural interactions with sex in taste processing.

### Heterogeneity in orobehavioral responses to bitter tastes

The present analyses found a variable influence of CNO perturbation of PB-*Calca* neurons on orosensory avoidance behavior to bitter taste stimuli. The increased acceptance of quinine shown, on average, by hM4Di mice under CNO was dependent on stimulus intensity, with these mice emitting more licks to quinine at elevated (≥0.3 mM) but not reduced (0.1 mM) concentrations compared with mCherry controls. In contrast, hM4Di and mCherry mice administered CNO did not differ in their licking responses to the bitter tastant cycloheximide.

There are some cautions to consider for comparing the quinine and cycloheximide results, including the smaller number of animals available for the cycloheximide analysis. Importantly, mice included in all analyses showed at least bilateral mCherry labeling of neurons, indicative of expression of hM4Di:mCherry or mCherry alone, in the external lateral PB nucleus, which is densely populated by *Calca* cells (Huang et al., 2021). Fewer mice contributed to the analysis of cycloheximide data because not all tested met this criterion, which was assessed postmortem. While this approach aimed to ensure that analyzed data were derived from mice with successful viral transduction, it is possible that the cycloheximide tests may have comparably less power to detect a difference between mouse groups. Furthermore, the rising concentration steps of quinine and cycloheximide caused systematic reductions in licking, but it is unclear if these steps are perceived by mice as intensity-equivalent between stimuli.

On the other hand, the differential effect of CNO on quinine and cycloheximide avoidance associates with established functional differences between these stimuli. In rodents, oral presence of quinine, an ionic bitter taste stimulus (Frank et al., 2004; Hettinger et al., 2007; Travers and Geran, 2009), normally evokes robust electrophysiological responses in cranial nerve (CN) VII and CN IX (Dahl et al., 1997; Inoue et al., 2001; Danilova and Hellekant, 2003; Damak et al., 2006). CN VII and CN IX, respectively, supply rostral and caudal lingual taste bud fields and are evidenced to support different aspects of taste processing and behavior under certain conditions (Travers et al., 1987; Spector and Grill, 1992; Spector et al., 1997; St John and Spector, 1998; see also St John and Boughter, 2004). In contrast to quinine, the nonionic (Hettinger et al., 2007; Travers and Geran, 2009) bitter taste stimulus cycloheximide induces strong gustatory activity mainly in CN IX (Danilova and Hellekant, 2003; Damak et al., 2006; Hettinger et al., 2007). Differences in gustatory neural responses to cycloheximide and quinine also emerge in the rodent CNS, where these stimuli evoke partly overlapping but distinct neural population responses in brainstem structures that process taste (Geran and Travers, 2006; Wilson et al., 2012), including the PB nucleus (Geran and Travers, 2009; Li and Lemon, 2019; Li et al., 2022).

While the function of heterogeneity in central neural responses to quinine and cycloheximide remains unknown, it is curious if PB-*Calca* neurons participate in brain circuits that support distinctions in neural information between these stimuli. Caveats notwithstanding, the present results would support this concept given that perturbation of *Calca* neurons was followed by different results on orosensory guided behaviors to quinine and cycloheximide. Other PB neural types may also participate in these circuits, including Satb2-positive neurons found to contribute to quinine licking avoidance behaviors alongside *Calca* cells (Jarvie et al., 2021). Satb2-positive neurons populate ventral lateral and medial PB regions (Fu et al., 2019; Jarvie et al., 2021; Kalyanasundar et al., 2023) that are associated with gustatory processing and contain neurons that activate to bitter taste stimuli in B6 mice (Tokita et al., 2014; Tokita and Boughter, 2016; Kalyanasundar et al., 2023). Thus, *Calca* neurons appear to be only a component of a PB neural system that mediates bitter taste processing. Along this line, oral presence of cycloheximide stimulates taste-active neurons located in PB areas (Geran and Travers, 2009; Li and Lemon, 2019; Li et al., 2022) that are populated by *Calca* cells (Huang et al., 2021). In light of the present results, neural input concerning cycloheximide taste could speculatively engage PB-*Calca* neurons, but the actions of another neural class exert greater influence on taste reactions to cycloheximide. Further studies are needed to probe this hypothesis and could help shed light on the physiological significance of differences in neural sensitivity that appear between quinine and cycloheximide and other diverse bitter taste stimuli (Geran and Travers, 2006, 2009; Travers and Geran, 2009; Wilson et al., 2012). Furthermore, the available functional data indicate that only a fraction of the population of PB-*Calca* neurons may strongly excite during quinine consumption (Kang et al., 2022), suggesting there may be specializations among these neurons for taste.

### PB-*Calca* neurons and oral thermal preferences

We included brief-access tests with temperature-controlled water to examine how chemogenetic dampening of PB-*Calca* neurons influenced orosensory preferences toward innocuous stimuli. Our prior data show that when given a choice in a brief-access setting, water-restricted B6 mice will readily lick mild cool (15°C) water, at a near-maximal rate, but avoid innocuous (physiological) warm (35°C) water, with the latter eliciting reduced average licks per trial (Li et al., 2024). This phenomenon was replicated presently in both mCherry and hM4Di mice, which were indifferent in their licking preference for 15°C and avoidance of 35°C water under CNO (Fig. 3). This result implied that PB-*Calca* neurons do not participate in innocuous oral thermal preferences.

Notably, licking avoidance of innocuous warm fluids is context dependent. If 35°C water is the only fluid offered in a brief-access setting, water-restricted B6 mice will lick 35°C water at a near-maximal rate, like that evoked by room temperature water (Li et al., 2024). Relatedly, innocuous warm 40°C water stimulates more licking than room temperature water in rats performing in longer-term single-bottle test sessions with each temperature (Kay et al., 2020). This avidity to lick warm water when offered alone contrasts behavioral responses to singly proffered quinine solutions. Rodents can show reduced licking of quinine compared with room temperature water when quinine is the only fluid offered in extended (Yamamoto et al., 1985; Spector and St John, 1998) and short (Hsiao and Fan, 1993) duration consumption tests.

While challenging to directly compare behavioral differences across sensory modalities, the above would suggest that licking avoidance of quinine may reflect the actions of an orobehavioral process that has some functional distinction from that driving responses to innocuous oral temperatures. Accordingly, physiological/innocuous warm water, albeit less preferred than cooling, is unlikely to cause ingestive harm (Kay et al., 2020) whereas bitterness can reflect poison (Glendinning, 1994). That suppression of PB-*Calca* neurons presently appeared to lessen avoidance of quinine, but not oral warmth, agrees with the discussed role of this neural type in limiting harm (Palmiter, 2018). We caution, though, that there are discrepancies in sample sizes between the present quinine and thermal data, with the thermal tests potentially having less power due to lower mouse numbers. Nonetheless, other data also suggest PB-*Calca* neurons may differentially participate in innocuous thermal and bitter taste sensation. While quinine consumption can excite PB-*Calca* neurons, cutaneous warming does not unless rising temperatures leave the innocuous range and exceed noxious heat threshold above ∼45°C (Campos et al., 2018; Kim et al., 2024b).

### Variability in orosensory behaviors

The present analyses demonstrated notable variance in mouse orosensory responses to individual taste stimuli. For example, while the mean lick score to 0.3 mM quinine was significantly higher in hM4Di mice under CNO, a few of these animals showed no or only a mild increase in licks compared with the average mCherry control group response. In contrast, other hM4Di mice displayed substantially elevated responding, licking 0.3 mM quinine at water-like levels under CNO (Fig. 5*B*). The confidence interval for the difference between mouse groups demonstrated that a range of relatively small to larger increases in quinine licking by hM4Di mice are compatible with our data and could arise on replication (Fig. 5*B*).

For studies of this sort, it is important to consider that viral-based delivery of DREADDs to neural tissues may not affect all neurons of a targeted population (Smith et al., 2016). Moreover, DREADD effects on activity in transduced neurons are likely not complete such that CNO activation of hM4Di may only imbue modest hyperpolarization and dampen, but not fully silence, the cellular response (Roth, 2016; Smith et al., 2016). Variance in these parameters across hM4Di mice could have contributed to variance in the present behavioral effects that followed chemogenetic perturbations. Yet mCherry mice showed reduced but marked variance in licking responses in some cases (Fig. 5*B*), suggesting some of the present behavioral variance may reflect other physiological or experimental parameters not considered. What is more, some brains examined here showed evidence of viral leakage adjacent to the PB area with sparse mCherry expression observed in the cerebellum, which also contains CGRP-positive cells (Warfvinge and Edvinsson, 2019). Cerebellar output can influence lick frequency (Bryant et al., 2010), although hM4Di and mCherry mice did not presently show differences in oromotor responding gauged by water licking measured in the presence and absence of CNO.

Nevertheless, the present studies aimed to test sizable numbers of experimental and control mice to accommodate variance in responses. As above, smaller animal numbers were available for some analyses because not all mice tested displayed bilateral mCherry labeling of neurons, indicative of expression of hM4Di:mCherry or mCherry alone, in the external lateral PB nucleus containing *Calca* cells (Huang et al., 2021).

### Concluding remarks

While there are limitations to this study, the results accord with extant data concerning taste processing and PB-*Calca* neuron function. Prior studies have implicated PB-*Calca* neurons with a role in quinine taste (Jarvie et al., 2021; Kang et al., 2022; Kim et al., 2024b). That distinct orobehavioral results emerged for quinine and cycloheximide during chemogenetic dampening of PB-*Calca* cells associates with known functional differences between these bitters, including differential responding to them by taste-active PB neurons (Geran and Travers, 2009; Travers and Geran, 2009; Li and Lemon, 2019; Li et al., 2022). Furthermore, the present evidence that PB-*Calca* neurons affect orosensory behaviors to both avoided quinine and appetitive sucrose agrees with data suggesting these neurons dually participate in opposing sensory/physiological valence functions (Kim et al., 2024a,b). Our results imply that appetitive orosensory responses to sucrose influenced by PB-*Calca* neurons have significant dependence on sex. Future studies on the spatial and functional bases of brain cell types supporting taste and their relation to sex will help delineate gustatory coding.

## Synthesis

Reviewing Editor: David Schoppik, New York University Grossman School of Medicine Decisions are customarily a result of the Reviewing Editor and the peer reviewers coming together and discussing their recommendations until a consensus is reached. When revisions are invited, a fact-based synthesis statement explaining their decision and outlining what is needed to prepare a revision will be listed below. The following reviewer(s) agreed to reveal their identity: Anan Moran.

Major Concerns

1) The primary behavioral measure is the "mean lick" count, calculated using a 20% trimmed mean across trials, excluding those with low or high lick counts. This approach is unconventional for handling non-normal distributions (if that is the case). A more standard and interpretable method would be to use median-based statistics or other models suited for count data. Moreover, in the results, it appears that a 10% trimmed mean is sometimes reported instead. Ultimately, the rationale for trimming, beyond excluding zero-lick trials, is not clearly justified. This raises concerns about the robustness and interpretability of the reported effects.

2) We had issues with the controls, and the way the data were presented. It is unclear whether behavioral differences between mCherry and hM4Di mice were assessed in the absence of CNO (Figure 4). Including such a control would help isolate the effects of DREADD activation from baseline group differences. A second potential concern arises with the use of the no-CNO group. Although the authors appropriately use an mCherry control in most of the figures, in some cases, such as the data shown in figure 11, comparisons are made between the CNO and non-CNO conditions. It is unclear what exactly constitutes the no-CNO group in this context. If this group did not receive an intraperitoneal injection at all, this could present a confounding event. When comparing animals receiving CNO injections with animals not receiving injections, the injection itself (independent of the drug's pharmacological action) could influence behavior, particularly under water deprivation. Injected fluid contributes to hydration, which can alter drinking behavior or motivation, especially in the context of a brief-access test. Furthermore, handling and injection stress could affect behavior independently of the drug. For these reasons, it is advisable to include a matched vehicle-injected control group to account for these potential differences in hydration and stress exposure. Clarifying this aspect of the experimental design and, if applicable, considering a vehicle control would strengthen the interpretation of the CNO-specific effects.

3) The study infers a functional role for Calca-expressing neurons in taste-guided behavior based solely on their inhibition. However, this approach does not distinguish whether these neurons are uniquely involved or simply part of the PBN neuronal population contributing to the behavior. For instance, suppose Calca-neurons are 50% of the total PBN neurons. If inhibiting any 50% of the neurons will give similar results, it means that Calca-neurons have no specific role in the behavior tested. To strengthen this claim, the authors could include control experiments targeting non-Calca neurons or randomly selected PBN neurons to demonstrate specificity. Furthermore, while quinine avoidance and sucrose preference were modulated by Calca neuron suppression, the aversiveness of cycloheximide and warm water -- which the authors' own data and the prior literature indicate are aversive stimuli to water-deprived mice -- was not affected by Calca neuron manipulation.

Ultimately, we feel that these findings seem more consistent with the role of PB Calca neurons in the differential modulation of fluid consumption dependent on taste stimulus, rather than in the general coding of valence across the aversive and appetitive domains. A more tempered interpretation along these lines would better align the conclusions with the data presented.

4) The conclusions drawn from the cycloheximide experiments should be tempered due to the small number of animals tested. This limitation should be explicitly discussed, and the interpretation of differences between quinine and cycloheximide should be presented with appropriate caution. This is especially important considering the interpretation that PB-Calca neurons are differentially engaged in processing distinct aversive bitterants. It remains possible that the absence of an observed effect with cycloheximide maybe due to the smaller sample size in that condition, rather than a definitive lack of involvement of PB-Calca neurons. Clarifying this possibility would further strengthen the manuscript's conclusions.

5) The results section includes intriguing data examining the impact of Calca neuron suppression on water intake at different temperatures, a factor previously demonstrated (including by the authors themselves in figure 3) to have distinct aversive "hedonic" significance. However, these results are not subsequently addressed in the discussion, leaving this reader uncertain about: (1) their relevance to the central conclusions, and (2) whether interpretative concerns related to sample size (similar to the one noted in comment 4) may apply, especially when compared to the larger datasets for quinine and sucrose. Clarifying the necessity or interpretation of these temperature-based findings would greatly enhance the coherence and overall strength of the manuscript.

6) For Figure 1, it would be helpful if the authors could provide the counterstaining alongside the mCherry signal and the reconstructed borders of the brain regions.

Minor Points

1) The manuscript would benefit from a more detailed discussion of what type of information PB-Calca neurons might encode-e.g., taste identity, hedonic value, or motivational salience. Additionally, the observed sex differences in sucrose preference warrant further exploration. Are these differences due to hormonal modulation, circuit-level divergence, or other factors?

## Author Response

Synthesis Statement for Author (Required):

Major Concerns 1) The primary behavioral measure is the "mean lick" count, calculated using a 20% trimmed mean across trials, excluding those with low or high lick counts. This approach is unconventional for handling non-normal distributions (if that is the case). A more standard and interpretable method would be to use median-based statistics or other models suited for count data. Moreover, in the results, it appears that a 10% trimmed mean is sometimes reported instead. Ultimately, the rationale for trimming, beyond excluding zero-lick trials, is not clearly justified. This raises concerns about the robustness and interpretability of the reported effects. << Response >> Thank you for this comment. We have expanded on our rationale for using trimmed means in the revised manuscript. The edits reflect the following:

Mean lick counts were estimated using trimmed means because stimulus lick count distributions observed for each mouse were not always normally distributed (p < 0.05, Jarque-Bera goodness-of-fit test). Importantly, trimmed means were not used to exclude zero-lick, or non-sampled, trials: these were simply ignored and not counted in any calculations. "Mean licks" was based only on trials where at least 1 lick was recorded, which reflects engagement with the sipper tube. Our edits aimed to further clarify this in the text. We apologize for the confusion.

We used trimmed mean statistics because skew, outliers, and unequal group variances were present for some group comparisons. Trimmed means, like medians, are "robust" in that they can show resistance to skew and outliers. Moreover, trimmed mean statistics can retain data magnitude information (e.g., lick count), making them appropriate for lick response data. Finally, trimmed mean statistics also retain power. Yuen's trimmed t, as used presently, shows only slightly less power than a Student's t under normality and offers advantages when data are skewed. We now refence this in the revised text.

In contrast, non-parametric statistics that work with median-based hypotheses can discard data magnitude information (e.g., lick count) by converting data points to ranks. Non-parametric tests also typically have less power than their parametric counterparts.

The review is correct that trimmed means and robust statistics (e.g., Yuen's test) are new to the gustatory behavioral literature. Yet robust statistics offer advantages over conventional tests faced with non-normal and heteroscedastic data. These situations are common in gustatory neurobiology and lickometry. Thus, we chose to use trimmed means and robust tests where possible, in addition to estimation statistics. We have also recently published (2024) other brief-access behavioral data that were based on trimmed means.

We have revised Figure 3 and 11 to use 20% trimmed means for sample locations. Interpretations do not change.

2) We had issues with the controls, and the way the data were presented. It is unclear whether behavioral differences between mCherry and hM4Di mice were assessed in the absence of CNO (Figure 4). Including such a control would help isolate the effects of DREADD activation from baseline group differences. << Response >> We did not test bitter quinine (Figure 4), or bitter cycloheximide, under no-CNO conditions. Only 4 day with-CNO tests, with between-subjects control, were performed with bitters to reduce test exposure to these stimuli and related non-specific effects. Water restriction, needed to motivate sampling of bitter-tasting fluids, may cause cumulative effects on behavior across sequential test days (Spector and St. John, 1998). Bitter taste exposure can also elevate salivary proteins that affect bitter taste sensitivity in rodents, with this effect arising in about 4 days (Martin et al. 2023). Other data reveal rapid modifications of the salivary proteome after quinine taste stimulation (Quintana et al., 2009).

Our design was between-subjects, where hM4Di carrier and non-carrier (mCherry) control mice received building exposure to bitter tastes and CNO in synchrony. This aimed to evenly spread potential confounds of variable thirst motivation, for example, between control and experimental study groups. Our synchronous use of mCherry mice controlled for the intracranial viral microinjection procedure, CNO administration, time of testing, and bitter taste exposure. We have added further clarification on these points to the methods section of the manuscript. Note that water restriction was not used during tests with sucrose, as addressed in the response to the next point.

We assessed but did not find (p > 0.05) differences in baseline licking between mCherry and hM4Di mice, examined prior to the onset of any testing (Figure 2). Furthermore, our analyses of standardized data (lick scores and lick ratios) controlled for potential individual differences in licking if present during tests.

A second potential concern arises with the use of the no-CNO group. Although the authors appropriately use an mCherry control in most of the figures, in some cases, such as the data shown in figure 11, comparisons are made between the CNO and non-CNO conditions. It is unclear what exactly constitutes the no-CNO group in this context. If this group did not receive an intraperitoneal injection at all, this could present a confounding event. When comparing animals receiving CNO injections with animals not receiving injections, the injection itself (independent of the drug's pharmacological action) could influence behavior, particularly under water deprivation. Injected fluid contributes to hydration, which can alter drinking behavior or motivation, especially in the context of a brief-access test. Furthermore, handling and injection stress could affect behavior independently of the drug. For these reasons, it is advisable to include a matched vehicle-injected control group to account for these potential differences in hydration and stress exposure. Clarifying this aspect of the experimental design and, if applicable, considering a vehicle control would strengthen the interpretation of the CNO-specific effects. << Response >> Apologies - we did not adequately describe the no-CNO condition in the first draft. In the revision, we clarify that on the no-CNO test days for sucrose, each mouse received a simulated i.p. injection procedure prior to their brief-access session. To do this, mice were scruffed by the neck skin behind the ears. While scruffed, mice were elevated by the experimenter, and a plastic syringe (without needle) was applied to abdominal skin medial to the inner thigh to mimic handling that would be received during an i.p. injection. Mice were then released into their home cage. Brief access testing began about 30 minutes later. This procedure aimed to control for effects of experimenter handling and related stress on mice across sucrose sessions conducted with and without CNO injections.

Notably, mice were not water restricted during all with- and no-CNO test sessions with sucrose. Thus, thirst would not be expected to be a major influence on licking in our sucrose tests. Food restriction was also not used in our studies. Mice presumably licked sucrose only for its hedonic (preference) property, as evidenced by increasing brief-access licks as sucrose concentration increased. We added further clarification on this to the methods.

We did observe that female mCherry mice gave significantly more licks to sucrose during the no-CNO compared to with-CNO tests (Figure 11). This could agree with lower hydration during the no-CNO condition due to absence of an injection, in addition to other possibilities. Yet mice were not water restricted during all with- and no-CNO test sessions with sucrose, suggesting thirst would not play a major role in licking during the present sucrose tests. Moreover, experimental hM4Di mice did not show increased licks to sucrose during the no-CNO tests, with hM4Di males displaying lingering attenuated sucrose preference without CNO. This finding implies that even with potential influences of injection hydration removed, hM4Di males did not recover control-like preferences for sucrose during no-CNO tests. This continues to suggest there is some lingering effect of CNO that persists after CNO administration is discontinued. We have edited our discussion to reflect this.

3) The study infers a functional role for Calca-expressing neurons in taste-guided behavior based solely on their inhibition. However, this approach does not distinguish whether these neurons are uniquely involved or simply part of the PBN neuronal population contributing to the behavior. For instance, suppose Calca-neurons are 50% of the total PBN neurons. If inhibiting any 50% of the neurons will give similar results, it means that Calca-neurons have no specific role in the behavior tested. To strengthen this claim, the authors could include control experiments targeting non-Calca neurons or randomly selected PBN neurons to demonstrate specificity. Furthermore, while quinine avoidance and sucrose preference were modulated by Calca neuron suppression, the aversiveness of cycloheximide and warm water -- which the authors' own data and the prior literature indicate are aversive stimuli to water-deprived mice -- was not affected by Calca neuron manipulation.

Ultimately, we feel that these findings seem more consistent with the role of PB Calca neurons in the differential modulation of fluid consumption dependent on taste stimulus, rather than in the general coding of valence across the aversive and appetitive domains. A more tempered interpretation along these lines would better align the conclusions with the data presented. << Response >> Thank you for these comments. We edited the text to further clarify that, based on prior data, Calca neurons may only be a component of the PB neuronal population contributing to the measured behaviors. This is evidenced by data that implicate other PB neural types in taste (e.g., Satb2 neurons) and the presence of gustatory neurons in diverse subnuclei of the PB area, as discussed in the revision. Moreover, prior imaging data suggest that quinine consumption robustly activates only a fraction of PB-Calca cells, implying there may be specializations among these neurons in taste processing.

While we do agree that there are additional studies on PB neurons in taste to perform, we reason that such effort would be beyond the scope of the present focus on Calca neurons. As highlighted above and further in the manuscript, other work has addressed this to some extent, identifying other PB cell type populations (Satb2) that operate alongside Calca cells during gustatory processing. The literature would suggest that perturbation of random PB neurons may lead to difficult to interpret effects due, in part, to potential heterogeneous functions of the affected cells. Variability in the types of impacted neurons affected by non-selective perturbation would also be expected across mice.

Prior data that bear specifically on the function of PB-Calca neurons suggest these cells contribute valence information to sensory signals. Previous studies show PB-Calca neurons are activated by valence-aligned inputs from diverse systems and body areas, including craniofacial regions, interoception, spinal pain, and itch. Other data have suggested these cells can also respond to opposing valence cross-modal stimuli that engage different body areas. This feature of these neurons would imply they do not register stimulus location or identity but the value of the input to an animal. Taste appears to be part of the sensory repertoire signaled by at least some PB-Calca cells, as evidenced by prior and the present data. Along this line, mice were not water-, or food-, restricted during the present tests with sucrose. Thus, it was presumed that the preferred hedonic tone of sucrose, and not thirst or hunger, was the primary motivator of licking to this stimulus, with reduced licks to sucrose noted in male mice during perturbation of PB-Calca neurons.

We edited the text to further clarify that while there are limitations to the present work, there are empirical data that suggest the neural processing of quinine and cycloheximide taste differs. This associates with the different results for these stimuli that followed perturbation of PB-Calca neurons. We also now discuss data that imply avoidance of oral warmth may have some functional differences with bitter taste avoidance, which is also in accord with the different results observed for these stimuli. Finally, we have tempered the conclusions to focus only on taste and oral sensation. Other published data have suggested a broader role for PB-Calca neurons in valence signaling, as above.

4) The conclusions drawn from the cycloheximide experiments should be tempered due to the small number of animals tested. This limitation should be explicitly discussed, and the interpretation of differences between quinine and cycloheximide should be presented with appropriate caution. This is especially important considering the interpretation that PB-Calca neurons are differentially engaged in processing distinct aversive bitterants. It remains possible that the absence of an observed effect with cycloheximide maybe due to the smaller sample size in that condition, rather than a definitive lack of involvement of PB-Calca neurons. Clarifying this possibility would further strengthen the manuscript's conclusions. << Response >> Done. We have expanded the discussion to address the limitations of the cycloheximide results.

5) The results section includes intriguing data examining the impact of Calca neuron suppression on water intake at different temperatures, a factor previously demonstrated (including by the authors themselves in figure 3) to have distinct aversive "hedonic" significance. However, these results are not subsequently addressed in the discussion, leaving this reader uncertain about: (1) their relevance to the central conclusions, and (2) whether interpretative concerns related to sample size (similar to the one noted in comment 4) may apply, especially when compared to the larger datasets for quinine and sucrose. Clarifying the necessity or interpretation of these temperature-based findings would greatly enhance the coherence and overall strength of the manuscript. << Response >> Thank you, we further discuss the significance of the thermal data, which may reflect a functional difference compared to quinine avoidance.

6) For Figure 1, it would be helpful if the authors could provide the counterstaining alongside the mCherry signal and the reconstructed borders of the brain regions. << Response >> Done.

Minor Points 1) The manuscript would benefit from a more detailed discussion of what type of information PB-Calca neurons might encode-e.g., taste identity, hedonic value, or motivational salience. Additionally, the observed sex differences in sucrose preference warrant further exploration. Are these differences due to hormonal modulation, circuit-level divergence, or other factors? << Response >> Thank you. As above, we have included more information on the function of Calca neurons in the introduction and discussion. We also expand discussion on sex differences in the discussion.
